# NPRC deletion mitigated atherosclerosis by inhibiting oxidative stress, inflammation and apoptosis in ApoE knockout mice

**DOI:** 10.1038/s41392-023-01560-y

**Published:** 2023-08-09

**Authors:** Cheng Cheng, Jie Zhang, Xiaodong Li, Fei Xue, Lei Cao, Linlin Meng, Wenhai Sui, Meng Zhang, Yuxia Zhao, Bo Xi, Xiao Yu, Feng Xu, Jianmin Yang, Yun Zhang, Cheng Zhang

**Affiliations:** 1 National Key Laboratory for Innovation and Transformation of Luobing Theory, Key Laboratory of Cardiovascular Remodeling and Function Research, Chinese Ministry of Education, Chinese National Health Commission and Chinese Academy of Medical Sciences, Department of Cardiology, Qilu Hospital of Shandong University, Jinan, China; 2 Department of Cardiology, Shengjing Hospital of China Medical University, Shenyang, Liaoning Province 110004 China; 3 Department of Traditional Chinese Medicine, Qilu Hospital, Cheeloo College of Medicine, Shandong University, Jinan, 250012 Shandong China; 4 Department of Epidemiology, School of Public Health, Cheeloo College of Medicine, Shandong University, Jinan, China; 5 Key Laboratory Experimental Teratology of the Ministry of Education, Department of Physiology, School of Basic Medical Sciences, Cheeloo College of Medicine, Shandong University, Jinan, China; 6 Department of Emergency Medicine, Chest Pain Center, Shandong Provincial Clinical Research Center for Emergency and Critical Care Medicine, Qilu Hospital, Shandong University, Jinan, China; 7 Cardiovascular Disease Research Center of Shandong First Medical University, Central Hospital Affiliated to Shandong First Medical University, Jinan, China

**Keywords:** Cardiology, Pathogenesis

## Abstract

Previous studies suggested a beneficial effect of natriuretic peptides in animal models of cardiovascular disease, but the role of natriuretic peptide receptor C (NPRC) in the pathogenesis of atherosclerosis (AS) remains unknown. This study was designed to test the hypothesis that NPRC may promote AS lesion formation and instability by enhancing oxidative stress, inflammation, and apoptosis via protein kinase A (PKA) signaling. ApoE^−/−^ mice were fed chow or Western diet for 12 weeks and NPRC expression was significantly increased in the aortic tissues of Western diet-fed mice. Systemic NPRC knockout mice were crossed with ApoE^−/−^ mice to generate ApoE^−/−^NPRC^−/−^ mice, and NPRC deletion resulted in a significant decrease in the size and instability of aortic atherosclerotic lesions in ApoE^−/−^NPRC^−/−^ versus ApoE^−/−^ mice. In addition, endothelial cell-specific NPRC knockout attenuated atherosclerotic lesions in mice. In contrast, endothelial cell overexpression of NPRC aggravated the size and instability of atherosclerotic aortic lesions in mice. Experiments in vitro showed that NPRC knockdown in human aortic endothelial cells (HAECs) inhibited ROS production, pro-inflammatory cytokine expression and endothelial cell apoptosis, and increased eNOS expression. Furthermore, NPRC knockdown in HAECs suppressed macrophage migration, cytokine expression, and phagocytosis via its effects on endothelial cells. On the contrary, NPRC overexpression in endothelial cells resulted in opposite effects. Mechanistically, the anti-inflammation and anti-atherosclerosis effects of NPRC deletion involved activation of cAMP/PKA pathway, leading to downstream upregulated AKT1 pathway and downregulated NF-κB pathway. In conclusion, NPRC deletion reduced the size and instability of atherosclerotic lesions in ApoE^−/−^ mice via attenuating inflammation and endothelial cell apoptosis and increasing eNOS expression by modulating cAMP/PKA-AKT1 and NF-κB pathways. Thus, targeting NPRC may provide a promising approach to the prevention and treatment of atherosclerosis.

## Introduction

Cardiovascular disease caused by atherosclerosis (AS) has become the major cause of human mortality worldwide leading to ≈17.6 million deaths annually.^1^ Although several risk factors such as dyslipidemia and inflammation have been causally linked to the pathogenesis of AS, the exact mechanism underlying the initiation and development of AS is not completely understood.^2–4^

To discover susceptible genes and explore a genetic marker of coronary artery disease (CAD) in the Chinese Han population, we recently performed a genome-wide association study in 3363 CAD patients and 3148 controls, and found 6 novel loci in natriuretic peptide receptor C (NPRC), which showed a significant association with CAD even after adjustment for traditional risk factors of CAD, suggesting that NPR-C gene SNPs significantly contribute to CAD susceptibility in the Chinese Han population.^5^ However, the molecular mechanism underlying the association between NPRC gene polymorphism and CAD is unclear.

Natriuretic peptides (NPs) are important molecules to maintain cardiovascular function, which include atrial natriuretic peptide (ANP), brain natriuretic peptide (BNP), and C-type natriuretic peptide (CNP).^6^ All NPs are synthesized in the human body in the form of "prohormone". The human ANP encoding gene, nppa, is first transcribed and translated into a 151-amino acid-length pre-proANP, which is cut to form a 126-amino acid-length proANP and stored in the atrium in granular form. Atrial wall pulling can stimulate the atrial secretion of stored proANP, which is rapidly cleaved by the serine protease “corin” on the cell membrane to produce an active fragment of 28 amino acids, exerting biological efficacy.^7^ The coding gene nppb of BNP is first transcribed and translated to form a pre-proBNP of 134 amino acids, and further forms a proBNP of 108 amino acids. After cutting, proBNP formed an active fragment of 32 residues at the C-end and an inactive fragment of 76 residues at the N-end. Although in physiological conditions BNP is co-stored with ANP in atrial granules at low concentrations, the primary source of BNP is synthesized and secreted by cardiomyocytes after the ventricular wall is stretched.^8^ CNP mainly exists in the central nervous system, vascular endothelial cells, and chondrocytes. Its encoding gene nppc is transcribed and translated into pre-proCNP of 126 amino acids length, and then cut into proCNP of 103 amino acids. The latter is cut by serine protease “furin” to form two active forms. They are 53 amino acid length CNP (CNP-53) and 22 amino acid length CNP (CNP-22). Unlike ANP and BNP, CNP is mainly synthesized and released by vascular endothelial cells in response to TNF-α, TGF-β, or shear forces.^9^ NPRC, a member of the natriuretic peptide system, is recognized as a clearance receptor of atrial natriuretic peptide (ANP), brain natriuretic peptide (BNP), and c-type natriuretic peptide (CNP), which acts through endocytosis and lysosomal degradation to modulate their physiological function and maintain the balance of the natriuretic peptide system.^10^ ANP and BNP are mainly secreted by ventricular and atrial myocytes,^11^ respectively, whereas CNP is primarily secreted by vascular endothelial cells.^12^

In the normal arterial wall, NPRC is mostly expressed in vascular endothelial cells, and less in vascular smooth muscle cells (VSMCs).^13^ Unlike natriuretic peptide receptor A (NPRA) and natriuretic peptide receptor B (NPRB), which are guanylyl cyclase receptors, NPRC lacks guanylyl cyclase activity but the cytoplasmic domain of NPRC constitutes guanine nucleotide regulatory protein (Gi) activator sequences that inhibit adenylyl cyclase activity.^14,15^ Recent studies found a notable difference in the affinity of natriuretic peptides for their receptors, and the rank order of affinities is ANP > BNP ≫ CNP for NPRA, CNP ≫ ANP > BNP for NPRB, and BNP > CNP > ANP for NPRC.^16^ Thus, ANP and BNP are mostly inclined to bind to NPRA, and NPRB is the most favored partner for CNP. By comparison, NPRC has a similar affinity to ANP, BNP, and CNP.^17^ It has been reported that CNP stimulates bone growth through CNP/NPRB signaling pathway.^18^ Similarly, the same bone overgrowth phenotype is also manifest in NPRC^−/−^ mice,^19^ probably due to an increased serum level of CNP in NPRC^−/−^ mice. However, the role of NPRC in atherosclerosis is controversial. Although earlier studies showed that increased NPRC expression was associated with instability of atherosclerotic plaques,^20,21^ a recent study found that CNP plays a major role in maintaining vascular homeostasis in atherosclerosis.^22^ More recently, binding of CNP with NPRC has been found to promote angiogenesis and vascular remodeling in response to ischemia via activating Gi, ERK1/2, and phosphoinositide 3-kinase γ/AKT signaling pathways.^23^ Thus, it remains elusive whether NPRC exerts a beneficial or harmful effect on AS lesions and what mechanism is involved in these effects.

In the present study, we hypothesized that NPRC may promote AS lesion formation and instability by enhancing oxidative stress, monocyte/macrophage migration and phagocytosis, endothelial cell apoptosis, and release of inflammatory mediators via protein kinase A (PKA) signaling. A series of experiments in vivo and in vitro were performed to test this hypothesis.

## Materials and methods

### Study animals

Eight-week-old apolipoprotein E-knockout (ApoE^−/−^) male mice were purchased from Beijing Viewsolid Biotechnology Co. LTD (Beijing, China). The experiments in vivo were consisted of four parts (Supplementary Fig. 1). In the first part of in vivo experiments, 20 ApoE^−/−^ mice, kept on a 12-h light/12-h dark cycle at 22 °C room temperature, were randomly divided into two groups (*n* = 10 per group), one group fed with a high fat and cholesterol Western diet (TP28521, Teluofei, China) and another fed with chow diet (LAD0011, Teluofei, China) for 12 weeks. In the second part of in vivo experiments, NPRC^−/−^ mice were constructed using CRISPR technology by Beijing Viewsolid Biotechnology Co. LTD (Beijing, China) from C57BL/6 background mice. According to the targeted specific hits reported by Matsukawa N,^24^ we built frameshift mutation at exon1(aa1-252) in conservative functional region (PBP1_NPRC_like) using CRISPR/Cas9 gene editing technique, which resulted in frameshift mutations in the whole transcription products of NPRC and achieved the purpose of protein function inactivation. After designing five CRISPR targets, we tested the efficiency of spCas9/gRNA and selected two targets: TCTCGGCGTGCGTGCTGCTGG and GGCTGGCGGCGCGAGCAGCGG. The RNAs transcribed in vitro from the above two gRNAs were microinjected into fertilized ovum along with spCas9 protein to derive expected mouse pups after screening. The genotyping of the NPRC^−/−^ mice was confirmed through agarose gel electrophoresis of the PCR fragments in the CRISPR-targeting region amplified from genomic DNA isolated from mouse tails with the following primers: Forward 5′-TTGGCGAGTTACTGAAGG-3′ and Reverse 5′-CGGTCCACAAGACTGAAG-3′. Cycling conditions were: 94 °C for 2 min, 98 °C for 10 s, 60 °C for 30 s, and 68 °C for 20 s for 35 cycles, and 68 °C for 10 min. Then we crossed ApoE^−/−^ mice with NPRC^−/−^ mice to derive ApoE^−/−^ NPRC^−/−^ mice. In the second part of in vivo experiments, 50 mice were randomly divided into two groups: ApoE^−/−^ group and littermates ApoE^−/−^NPRC^−/−^ group (*n* = 25 per group), and kept on a 12-h light/12-h dark cycle at 22 °C room temperature. These mice were fed with a high fat and cholesterol Western diet (TP28521, Teluofei, China) and water was freely available for 12 weeks. In the third part of in vivo experiments, B6/JGpt-Npr3em1Cflox/flox mice were crossed with B6/JGpt-Tek^em1Cin(iCre)^ mice (GemPharmatech Co., Ltd, Nanjing, China) to derive vascular endothelial cell-specific NPRC knockout (NPRC^ecKO^) mice. The genotyping of the NPRC^ecKO^ mice was confirmed through agarose gel electrophoresis of the PCR fragments in the CRISPR -targeting region amplified from genomic DNA isolated from mouse tails with the following Flox primers: Forward: 5′-GATCCAGAATCCAACTGAGTAGCATG-3′ and Reverse 5′-ACAGGAGCCAACAACACAGACACAA -3′. Cycling conditions were: 95 °C for 5 min, and 95 °C for 30 s, 58 °C for 30 s, and 72 °C for 30 s for 40 cycles, and 72 ˚C for 3 min. KI primers: H11-Tf3: 5′-GGGCAGTCTGGTACTTCCAAGCT-3′ and Tek-tR1: 5′-CTTGATTCACCAGATGCTGAGGTTA-3′. Cycling conditions were: 95 °C for 5 min, and 98 °C for 30 s, 65 °C (−0.5 ˚C/cycle) for 30 s and 72 °C for 40 s for 20 cycles, and 98 °C for 30 s, 55 °C for 30 s and 72 °C for 45 s for 20 cycles, and 72 ˚C for 5 min. WT primers: H11-tF3: 5′-GGGCAGTCTGGTACTTCCAAGCT-3′ and H11-tR3: 5′-ATATCCCCTTGTTCCCTTTCTGC-3′. Cycling conditions were: 95 °C for 5 min, and 98 °C for 30 s, 65 °C (−0.5 °C/cycle) for 30 s and 72 °C for 40 s for 20 cycles, and 98 °C for 30 s, 55 °C for 30 s and 72 °C for 45 s for 20 cycles, and 72 ˚C for 5 min. Twenty mice were randomly divided into two groups: NPRC^ecKO^ group and littermates NPRC^ecWT^ group (*n* = 10 per group), who were kept on a 12-h light/12-h dark cycle at 22 °C room temperature and fed with a high fat and cholesterol Western diet (TP28521, Teluofei, China) and water was freely available for 16 weeks after receiving an injection of 2.5 × 10^11^vg PCSK9 AAV through tail veins to induce a mouse model of atherosclerosis. In the fourth part of in vivo experiments, 30 mice were randomly divided into four groups: 10 ApoE^−/−^ mice and 5 ApoE^−/−^NPRC^−/−^ mice (Beijing Viewsolid Biotechnology Co. LTD) received an injection of 2.5 × 10^11^vg AAV9 containing ICAM-2 promotor-NPRC-EGFP-3flag-WPRE-SV40_polyA via tail veins to induce NPRC overexpression (OE) in vascular endothelial cells and generate ApoE^−/−OE^ and ANDK (ApoE and NPRC Double Knockout)^OE^ mice. In addition, 10 ApoE^−/−^ mice and 5 ApoE^−/−^NPRC^−/−^ mice received an injection of 2.5 × 10^11^vg AAV9 containing ICAM-2 promotor-EGFP-3flag-WPRE-SV40_polyA via tail veins to induce sham NPRC overexpression. These mice were fed with a high fat and cholesterol Western diet (TP28521, Teluofei, China) and water was freely available for 12 weeks.

Experimental animals were grouped in a randomized manner and investigators were blinded to the allocation of mouse groups when conducting experimental protocols. All animal experimental protocols were approved by the Ethics Committee and the Scientific Investigation Board of Shandong University Qilu Hospital, China according to the Animal Management Rules of the Chinese Ministry of Health.

### Body weight, blood pressure, and serum lipid levels

In the second part of in vivo experiments, body weight was measured at the end of the experiment by an electronic balance (Shimadzu Corp) in all mice. Blood pressure and heart rate were measured between 9:00 AM and noon at the end of the experiment by a noninvasive tail-cuff system (Softron BP-98A, Japan) in all mice who were trained to accommodate to the device, and derived values were reported as a mean of 3 consecutive measurements. Blood sample were collected from the left ventricle of mice through cardiac puncture prior to euthanasia with excess pentobarbital sodium. Serum lipids levels including total cholesterol (TC) (S03042, Leidu, China), low-density lipoprotein cholesterol (LDL-C) (S03029, Leidu, China), high-density lipoprotein cholesterol (HDL-C) (S03025, Leidu, China), very low-density lipoprotein cholesterol (VLDL-C) (CSB-E17089m, Cusabio, China), triglycerides (TG) (S03027, Leidu, China) and oxidized LDL (oxLDL) (CSB-E07933m, Cusabio, China) were measured by enzymatic assay in all mice.

### Meso Scale Discovery (MSD), EIA and cAMP assay

In the second part of in vivo experiments, serum samples were centrifuged at 3000 rpm for 15 min and 50 μL supernatant were collected. Based on electrochemical luminescence, the pre-coated MSD multifactor detection plate (K15069L-1) and MSD detector (QuickPlex SQ120) (MSD SCALE DISCOVERY, Rockville, MD, USA) were combined to measure serum levels of TNF-α, IL-6, MCP1, ICAM1, and VCAM1. Serum ANP, BNP, and CNP levels in mice were measured using the mouse ANP EIA kit (Raybiotech, EIAM-ANP-1), mouse BNP EIA kit (Raybiotech, EIAM-BNP-1) and mouse CNP EIA kit (Raybiotech, EIAM-CNP-1) following the manufacturer’s instructions. Signals were measured on a Biotek plate reader. cAMP levels in the aortic tissues were measured using the CyclicAMPXP chemiluminescent assay kit (Cell Signaling Technologies, 8019) following the manufacturer’s instructions. Signals were measured on a Biotek plate reader.

### Tissue preparation and histopathological analysis

In the second, third, and fourth part of in vivo experiments, aortic segments from the ascending aorta to the iliac bifurcation were detached from surrounding tissues, fixed in 4% paraformaldehyde overnight and then stained by Oil Red O to display aortic en face atherosclerotic lesions, while frozen sections of the aortic roots were stained for quantification of cross-sectional lesion area and analysis of lesion components. Cross sectional lesion area of the aortic roots was measured in 5 serial sections of H&E-stained slides (60 μm between sections). The first section was defined as one that captures all 3 leaflets of the aortic valve, and the 2 serial sections proximal and distal to the first section were selected for quantification. For measuring lipids deposited plaque, we stained lesion of aortic roots with Oil-Red O. Besides, frozen sections were stained by immunohistochemical reagents for monocyte/macrophage, vascular smooth muscle cell, and collagen, which were quantitated by the ratio of Moma2 positive, α-SMA positive and Sirius Red positive area to plaque area, respectively. The vulnerability index was calculated by the following formula: the relative positive staining area of (macrophages% + lipid%)/the relative positive staining area of (α-SMCs% + collagen%).^25^ Quantification of the aortic atherosclerotic lesions were performed by researchers who were blinded to group allocation.

### Immunohistochemistry

In the second, third, and fourth part of in vivo experiments, frozen sections of the aortic roots from atherosclerotic mice were hydrolyzed in ddH_2_O for 10 min, blocked with 5% non-immune goat serum for 30 min at 37 °C, and incubated with primary antibodies at 4 °C overnight. Meanwhile, we used non-immune IgG and solvent PBS as negative controls for the same species to exclude false positive results. The sections were then incubated with horseradish peroxidase-conjugated secondary antibodies for 60 min at 25 °C the next day, and DAB kit (ZSGB-Bio, Beijing) was used for color development. Hematoxylin were used to counterstain nuclei in immunohistochemical staining. For immunofluorescence staining, the sections were incubated with appropriate fluorescent secondary antibodies (Alexa Fluor594, ab150077, Abcam) for 30 min at 37 °C after washing with PBS. Cellular nuclei were stained with DAPI (ab104139, Abcam) for 5 min at 37 °C. The images were collected under a fluorescence microscope (Ni-E, Nikon, Japan) with an excitation wavelength.

### ROS assay

In the second, third, and fourth part of in vivo experiments, intracellular and tissue ROS levels were measured by dihydroethidium (DHE) fluorescence. Briefly, cells were incubated with 10 μmol/L DHE for 30 min, then washed with serum-free DMEM for three times. Finally, the cells were counterstained with DAPI and photographed via laser scanning confocal microscopy (LSM 710, Zeiss). Frozen sections of the aortic roots were incubated with 10 μmol/L DHE for 1 h, and then washed with 1XPBS for three times.

### RNA-seq analysis of gene expression

In the second part of in vivo experiments, the entire aortas isolated from ApoE^−/−^ mice (*n* = 5) and NPRC^−/−^ApoE^−/−^ mice (*n* = 5) were used for RNA-seq by LC-Bio Technology (Hangzhou, China). A total of 1 μg RNA per sample extracted from aorta was used as input material for RNA sample preparations. The RNA integrity was assessed by Bioanalyzer 2100 (Agilent, CA, USA) with RIN number >7.0, and confirmed by electrophoresis with denaturing agarose gel. Poly (A) RNA is purified from 1 μg total RNA using Dynabeads Oligo (dT)25-61005 (Thermo Fisher, CA, USA) using two rounds of purification. Then, the poly(A) RNA was fragmented into small pieces using Magnesium RNA Fragmentation Module (NEB, cat.e6150, USA) under 94 °C for 5–7 min. Thereafter, the cleaved RNA fragments were reverse-transcribed to create the cDNA by SuperScript™ II Reverse Transcriptase (Invitrogen, cat. 1896649, USA), which were next used to synthesize U-labeled second-stranded DNAs with E. coli DNA polymerase I (NEB, cat.m0209, USA), RNase H (NEB, cat.m0297, USA) and dUTP Solution (Thermo Fisher, cat. R0133, USA). An A-base was then added to the blunt ends of each strand for ligation to the indexed adapters. Each adapter contains a T-base overhang for ligating the adapter to the A-tailed fragmented DNA. Single- or dual-index adapters are ligated to the fragments, and size selection was performed with AMPureXP beads. After the heat-labile UDG enzyme (NEB, cat.m0280, USA) treatment of the U-labeled second-stranded DNAs, the ligated products are amplified with PCR. The average insert size for the final cDNA library was 300 ± 50 bp. Finally, we performed the 2 × 150 bp paired-end sequencing (PE150) on an Illumina Novaseq™ 6000 (LC-Bio Technology CO., Ltd., Hangzhou, China) following the manufacturer’s instruction. After removing the low-quality and undetermined bases, we used HISAT2 software to map reads to the genome. The mapped reads of each sample were assembled using StringTie with default parameters. After estimating the expression levels of all transcripts and analyzing expression levels for mRNAs, the differentially expressed mRNAs were selected with fold change >2 or fold change <0.5 with *p* value < 0.05 by R package edgeR, and KEGG enrichment to the differentially activated pathways were analyzed.

### Cell incubation

OxLDL were purchased from XieSheng (China), and forskolin and H89 were purchased from MCE (USA). Detailed information for reagents and antibodies were listed in Supplementary Table 1. Human aortic endothelial cells (HAECs) were obtained from ATCC and cultured in endothelial cell growth medium 2 (ECM, Promocell C22010). Mouse macrophage cell line (Raw 264.7, ATCC) was cultured in medium containing DMEM (Hyclone, USA) supplemented with 10% FBS (Gibco, USA), and 1% penicillin–streptomycin (Gibco, USA). Peritoneal macrophage was collected in induced peritonitis model as previously described.^26^ In brief, 1 ml sterile 3% meat broth was injected intraperitoneally into mice. Peritoneal cavity was lavaged with 30 mL non-serum DMEM for three times, and the cell suspension was collected 72 h later. After centrifugation at 1000 rpm for 5 min, the cells were re-suspended with fresh complete medium and cultured for later experiments. Human macrophages were obtained by THP-1 cell line (CL-0233, Procell, China) treated by 100 nM PMA (HY-18739, MCE, USA) for 24 h. HAECs were transfected by NPRC siRNA duplexes (si-NPRC) or NPRC overexpressing plasmid (oe-NPRC). HAECs transfected by nonsense siRNA duplexes (si-NC) or empty vector (oe-NC) were used as controls. After stimulation by oxLDL for 24 h, the supernatant was collected as endothelial conditional medium for following experiments.

### RNA interference

Lipofectamine RNAiMAX reagents (Invitrogen, USA) were used for transient transfection of siRNA duplexes into HAECs. The sequences of siRNAs with a high silencing efficiency were as follows: negative control (NC) sequences: sense 5′-UUCUCCGAACGUGUCACFUTT-3′, and antisense 5′-ACGUGACACGUUCGGAGAATT-3′. Human NPRC sequences were obtained from Ruibo (Guangzhou, China).

### Plasmid transfection

Lipofectamine 3000 reagents (Invitrogen, USA) were used for transfection of ICAM-2 promotor-NPRC-EGFP-3flag-WPRE-SV40_polyA or ICAM-2 promotor-EGFP-3flag-WPRE-SV40_polyA into HAECs.

### Migration assay

Transwell chemotactic assay was used for measuring macrophage migration. An equal number of Raw 264.7 cells were placed on the upper layer of the transwell (Corning 3422), and EC condition medium containing oxLDL (100 μg/ml) were added below the cell permeable membrane for 12 h. Cells migrating through the membrane were stained by crystal violet staining solution and the number of cells were counted in 5 random fields for each group under a microscope.

### Macrophage oxLDL engulf assay

Macrophage oxLDL engulf assay was performed as previously described.^27^ Raw264.7 cells (2 × 10^5^) were plated on 15 mm glass coverslips in 12-well plate and treated with EC condition medium containing oxLDL (100 μg/ml) for 36 h. The cells were fixed with 4% PFA for 10 min, stained with Oil-Red O for 10 min, and rinsed with 60% isopropanol for 5 seconds. The cell images were captured by Leica microscopy, and the ratio of the Oil-Red O positive staining area to the whole cell area was calculated to quantitate the extent of oxLDL engulf.

### Apoptosis assay

Apoptosis was detected by TUNEL staining with an in situ cell death detection kit (Roche). HAECs were fixed with 4% paraformaldehyde at room temperature for 1 h and washed by PBS for three times. Then, cells were permeabilized in PBS with 0.1% Triton X-100 and stained with TUNEL reaction mixture for 1 h at 37 °C. Finally, the cells were counterstained with DAPI and photographed via laser scanning confocal microscopy (LSM 710, Zeiss).

### RT–PCR

Total RNA was extracted from isolated aortic samples and HAECs by using the RNeasy mini kit (Qiagen, 74106, Germany). A PrimeScript RT reagent kit with gDNA Eraser (TaKaRa, Japan) was used to reverse-transcribe the RNA according to the manufacturer’s instructions. Reverse-transcription products of different samples were amplified by A Light Cycler480 instrument (Roche LightCycler480, Switzerland) using the SYBR Green PCR Master Mix (Roche, Switzerland) following the manufacturer’s instruction. Cycling conditions were: 95 °C for 10 min, and 95 °C for 15 s, 55 °C for 15 s, and 72 °C for 20 s for 40 cycles. The data were normalized by the level of β-actin expression in each individual sample. The 2^−ΔΔCt^ method was used to calculate relative expression changes. The primer sequences were listed in Supplementary Table 2.

### Western blot analysis

Western blot analysis was performed as described previously.^28^ Total protein was extracted from aorta tissues by using the Total Protein Extraction Kit (AT-022, Invent Biotechnologies, Plymouth, MN, USA) and from cells by using the Cell Lysis (Sigma, USA). Proteins were separated by SDS-PAGE, transferred to PVDF membrane, blocked by 5% BSA (Albumin from bovine serum) and incubated with primary antibodies overnight at 4 °C. Finally, transferred blots were displayed by using chemiluminescent reagent (WBKLS0500, Millipore, Germany) and exposed by a chemiluminescence instrument (GE, Amersham Imager 600RGB).

### Statistical analysis

All data were presented as mean ± SEM. All analyses were performed with GraphPad Prism 8 (GraphPad, San Diego, CA). Each experiment was repeated independently for a minimum five times. Shapiro–Wilk tests were used to assess normality assumption of the data distribution. For normally distribution, unpaired two-tailed Student’s t-tests were used to determine the statistical difference between two groups. To determine the statistical difference between multiple groups with one variable, one-way ANOVA followed by Dunnett or Tukey post hoc tests were performed. Two-way ANOVA followed by Tukey or Sidak post hoc tests was used to compare multiple groups with more than one variable. For data with a non-Normally distribution, a nonparametric statistical analysis was performed, and Kruskal–Wallis test followed by Dunn post hoc test was applied for multiple comparisons with one variable. In all statistical comparisons, a *P* value of <0.05 was considered statistically significant.

## Results

### NPRC expression is increased in atherosclerotic lesions in vivo

**Fig. 1:**
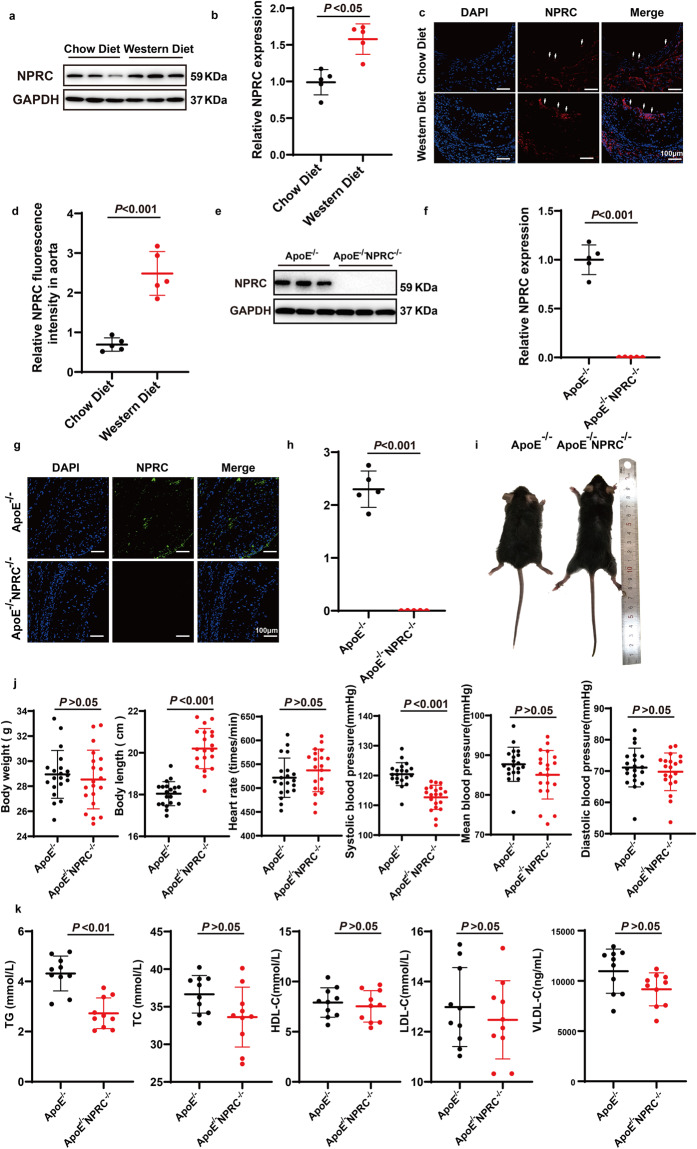
Comparison of NPRC expression between chow- and Western-diet-fed ApoE^−/−^ mice, and phenotypes between ApoE^−/−^ and ApoE^−/−^NPRC^−/−^ mice. **a** Representative Western blot images of NPRC expression in ApoE^−/−^ mice fed with chow and Western diet. **b** Quantification of NPRC expression in ApoE^−/−^ mice fed with chow and Western diet (*n* = 5 per group). **c** Representative immunofluorescence images of NPRC in aortic roots from ApoE^−/−^ mice fed with chow and Western diet (scale bar = 100 μm). **d** Quantification of NPRC immunofluorescent intensity in aortic roots from ApoE^−/−^ mice fed with chow and Western diet (*n* = 5 per group). **e** Representative Western blot images of NPRC expression in ApoE^−/−^ and ApoE^−/−^NPRC^−/−^ mice. **f** Quantification of NPRC expression in ApoE^−/−^ and ApoE^−/−^NPRC^−/−^ mice (*n* = 5 per group). **g** Representative immunofluorescence images of NPRC in ApoE^−/−^ and ApoE^−/−^NPRC^−/−^ mice (*n* = 5 per group). **h** Quantification of NPRC immunofluorescent intensity in aortic roots from ApoE^−/−^ and ApoE^−/−^NPRC^−/−^ mice (*n* = 5 per group). **i** Representative images of ApoE^−/−^ and ApoE^−/−^NPRC^−/−^ mice. **j** Quantification of body weight, body length, heart rate, and systolic, mean, and diastolic blood pressures in ApoE^−/−^ and ApoE^−/−^NPRC^−/−^ mice (*n* = 20 per group). **k** Quantification of serum levels of TG, TC, HDL-C, LDL-C, and VLDL-C (*n* = 10 per group). Normal distributions were tested by Shapiro–Wilk method. Unpaired two-tailed Student’s t tests were applied in (**c**), (**d**), (**f**), (**h**), (**j**), and (**k**)

In the first part of in vivo experiment, Western blot analysis demonstrated increased NPRC expression in the aortic tissues of ApoE^−/−^ mice fed with Western diet compared with those fed with chow diet (Fig. 1a, b). Similar results were observed in the aortic roots of the two groups of mice by immunofluorescence-staining of NPRC (Fig. 1c, d). The increased NPRC expression in atherosclerotic lesions suggests a potential role of NPRC in the regulation of atherosclerosis development.

### NPRC deletion attenuates atherosclerotic lesions in vivo

**Fig. 2:**
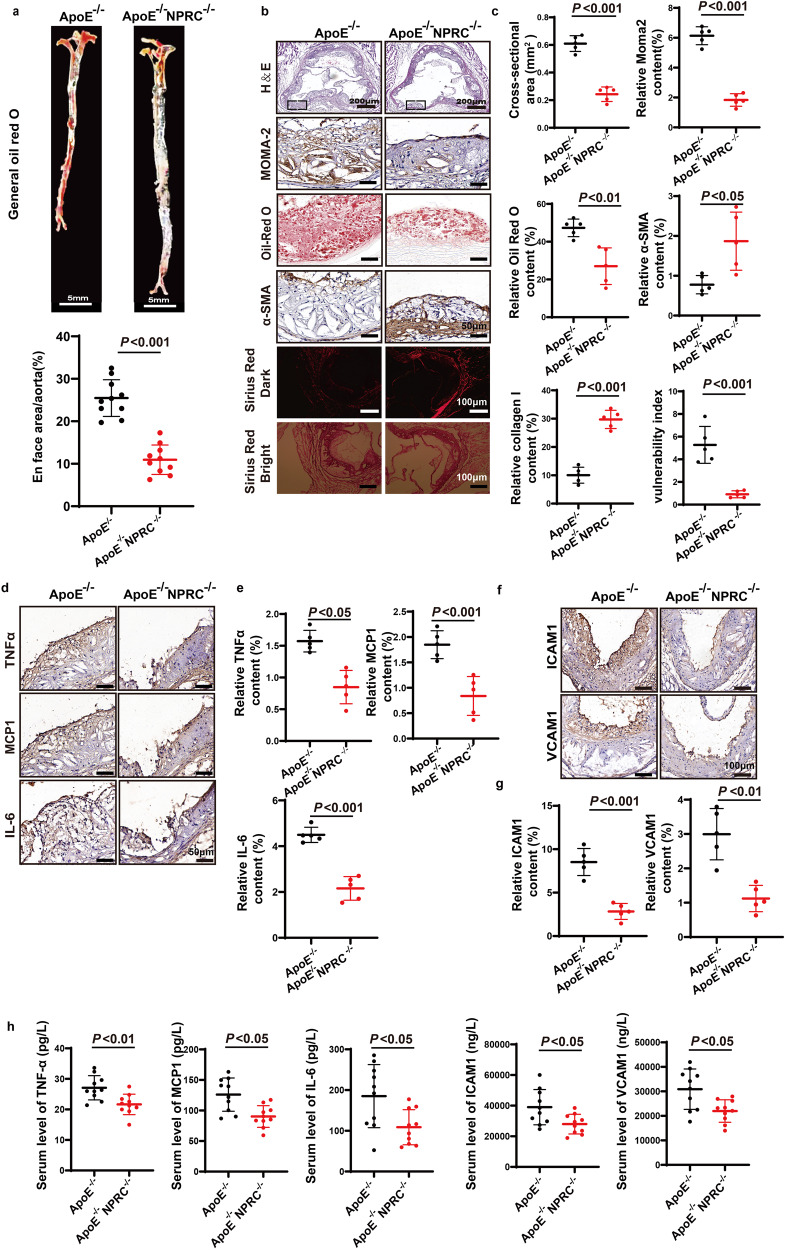
Comparison of atherosclerotic lesions and inflammatory cytokine expression between ApoE^−/−^ and ApoE^−/−^NPRC^−/−^ mice. **a** Representative images of Oil Red O staining of en face aorta (upper panel) and quantification of Oil red O positive staining area (lower panel) in ApoE^−/−^ and ApoE^−/−^NPRC^−/−^ mice (*n* = 10 per group) (scale bar = 5 mm). **b** Representative images of H&E staining (scale bar = 200 μm), Oil Red O staining (scale bar = 50 μm), Sirius red staining (scale bar = 100 μm) and immunohistochemical staining for Moma2 (scale bar = 50 μm) and α-SMA (scale bar = 50 μm) in the aortic tissues of ApoE^−/−^ and ApoE^−/−^NPRC^−/−^ mice. **c** Quantification of cross-sectional and en face aortic plaque area, and Moma2, α-SMA and collagen I positive staining area in ApoE^−/−^ and ApoE^−/−^ NPRC^−/−^ mice (*n* = 5 per group). **d** Representative images of immunohistochemical staining for TNFα, MCP1, and IL-6 in atherosclerotic lesions of ApoE^−/−^ and ApoE^−/−^NPRC^−/−^ mice (scale = 50 μm). **e** Quantification of TNFα, MCP1, and IL-6 positive staining area in atherosclerotic lesions of ApoE^−/−^ and ApoE^−/−^NPRC^−/−^ mice (*n* = 5 per group). **f** Representative images of immunohistochemical staining for ICAM1 and VCAM1 in atherosclerotic lesions of ApoE^−/−^ and ApoE^−/−^NPRC^−/−^ mice (scale = 100 μm). **g** Quantification of ICAM1 and VCAM1 positive staining area in atherosclerotic lesions of ApoE^−/−^ and ApoE^−/−^NPRC^−/−^ mice (*n* = 5 per group). **h** Serum levels of IL-6, MCP1, TNFα, ICAM1, and VCAM1 in ApoE^−/−^ and ApoE^−/−^NPRC^−/−^ mice measured by MSD (*n* = 10 per group). Normal distributions were tested by Shapiro–Wilk method. Unpaired two-tailed Student’s t tests were applied in (**a**), (**c**), (**e**), (**g**), and (**h**)

To explore the role of NPRC in atherosclerotic lesion formation, we generated ApoE^−/−^NPRC^−/−^ mice in the second part of in vivo experiments (Supplementary Fig. 2a). Deficient NPRC expression in aortas of ApoE^−/−^NPRC^−/−^ mice was confirmed by Western blot and immunofluorescence (Fig. 1e–h). ApoE^−/−^NPRC^−/−^ mice showed a bone-overgrowth phenotype, with a notable increase of body length (Fig. 1i, j) and aortic length (Fig. 2a) in comparison with littermate ApoE^−/−^ mice. There was no significant difference in body weight, heart rate, diastolic and mean blood pressure (Fig. 1j), and the serum levels of TC, LDL-C, HDL-C, and VLDL-C between ApoE^−/−^NPRC^−/−^ and littermate ApoE^−/−^ mice (Fig. 1k). However, systolic blood pressure and serum levels of TG were lower in ApoE^−/−^NPRC^−/−^ mice than their littermate ApoE^−/−^ mice, the latter of which was probably related to a thinner phenotype of ApoE^−/−^NPRC^−/−^ mice (Fig. 1j, k). Systemic deletion of NPRC resulted in a 63% reduction of atherosclerotic lesion area (*P* < 0.001) as demonstrated by Oil-red O staining of the en face aorta versus that of ApoE^−/−^ mice (Fig. 2a). Similarly, the cross-sectional area of atherosclerotic lesions in the aortic root was decreased by 60% (*P* < 0.001) as displayed by H&E staining in ApoE^−/−^NPRC^−/−^ mice versus ApoE^−/−^ mice (Fig. 2b, c). These results indicated that NPRC deletion attenuated atherosclerotic lesions in ApoE^−/−^NPRC^−/−^ mice relative to ApoE^−/−^ mice.

### NPRC deletion enhances stability of atherosclerotic lesions in vivo

Next, we analyzed the cellular composition of the atherosclerotic lesions by immunostaining of the aortic root in the second part of in vivo experiments. The relative content of MOMA2^+^-macrophage/monocytes and Oil-red O positive staining area in these lesions were reduced by 59% (*P* < 0.001) and 43% (*P* < 0.01), respectively, in ApoE^−/−^NPRC^−/−^ mice relative to the littermate ApoE^−/−^ mice, suggesting that the extent of inflammation and the lipid deposition in atherosclerotic lesions were substantially diminished by NPRC deletion. On the other hand, the relative content of smooth muscle cells as measured by alpha-smooth muscle actin positive staining area, and that of collagen as measured by Sirius-red positive stained area in atherosclerotic lesions were increased by 84% (*P* < 0.001) and 64% (*P* < 0.001), respectively, in ApoE^−/−^NPRC^−/−^ mice in comparison with the littermate ApoE^−/−^ mice (Fig. 2b, c). Consequently, the vulnerability index of atherosclerotic lesions was reduced by 53% (*P* < 0.001) in ApoE^−/−^NPRC^−/−^ mice versus the littermate ApoE^−/−^ mice (Fig. 2c). These results indicated that systemic NPRC deletion enhanced the stability of the aortic atherosclerotic lesions in ApoE^−/−^NPRC^−/−^ mice versus ApoE^−/−^ mice.

### NPRC deletion reduces pro-inflammatory cytokines in plasma and atherosclerotic lesions in vivo

To reveal the underlying molecular mechanisms of NPRC-deletion-mediated anti-atherosclerotic effects, we performed RNA high-throughput sequencing, using the whole aortas of ApoE^−/−^NPRC^−/−^ and ApoE^−/−^ mice (*n* = 5 in each group, Supplementary Fig. 3a) in the second part of in vivo experiments. The results showed that the top 10 pathways exhibiting the most prominent difference between ApoE^−/−^NPRC^−/−^ and ApoE^−/−^ mice in KEGG or GO pathway enrichment analysis were inflammation-related pathways (Supplementary Fig. 3a). In addition, the expression levels of pro-inflammatory cytokine including TNFα, MCP1, IL-6, ICAM1 and VCAM1 were lower in the aortic tissues of ApoE^−/−^NPRC^−/−^ mice than ApoE^−/−^ mice, as demonstrated by qPCR (Supplementary Fig. 3b), Western blot (Supplementary Fig. 3c, d) and immunohistochemical staining (Fig. 2d to g). Furthermore, serum levels of TNFα, IL-6 and MCP1, ICAM1, and VCAM1 were significantly lower in ApoE^−/−^NPRC^−/−^ mice than ApoE^−/−^ mice (Fig. 2h). These data indicated that NPRC deletion led to a substantial reduction of local and systemic inflammation in ApoE^−/−^ mice.

### Endothelial cell-specific NPRC knockout attenuates the size and instability of atherosclerotic lesions in vivo

**Fig. 3:**
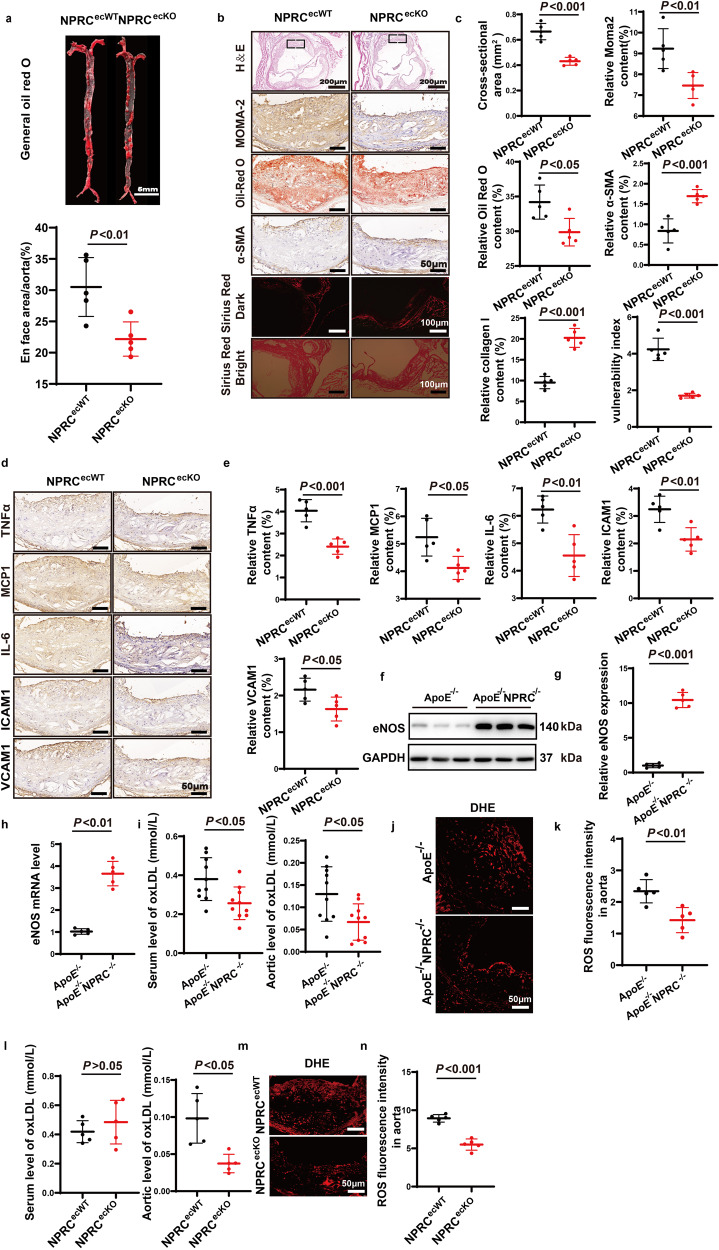
Comparison of atherosclerotic lesions, inflammatory cytokine expression, and oxidative stress between NPRC^ecWT^ and NPRC^ecKO^ mice. **a** Representative images of Oil Red O staining of en face aorta (upper panel) and quantification of Oil red O positive staining area (lower panel) in NPRC^ecWT^ and NPRC^ecKO^ mice (*n* = 5 per group) (scale bar = 5 mm). **b** Representative images of HE staining (scale bar = 200 μm), Oil Red O staining (scale bar = 50 μm), Sirius red staining (scale bar = 100 μm), and immunohistochemical staining for Moma2 (scale bar = 50 μm) and α-SMA (scale bar = 50 μm) in the aortic tissues of NPRC^ecWT^ and NPRC^ecKO^ mice. **c** Quantification of cross-sectional and en face aortic plaque area, and MOMA2, α-SMA and collagen I positive staining area in NPRC^ecWT^ and NPRC^ecKO^ mice (*n* = 5 per group). **d** Representative images of immunohistochemical staining for TNFα, MCP1, IL-6, ICAM1, and VCAM1 in atherosclerotic lesions of NPRC^ecWT^ and NPRC^ecKO^ mice (scale = 50 μm). **e** Quantification of TNFα, MCP1, IL-6, ICAM1, and VCAM1 positive staining area in atherosclerotic lesions of NPRC^ecWT^ and NPRC^ecKO^ mice (*n* = 5 per group). **f** Representative Western blot images of eNOS expression in aorta from ApoE^−/−^ and ApoE^−/−^NPRC^−/−^ mice (*n* = 5 per group). **g** Quantification of eNOS expression in aorta from ApoE^−/−^ and ApoE^−/−^NPRC^−/−^ mice (*n* = 5 per group). **h** Quantification of mRNA level of eNOS in aorta from ApoE^−/−^ mice and ApoE^−/−^NPRC^−/−^ mice (*n* = 5 per group). **i** Quantification of oxLDL level in serum and aorta from ApoE^−/−^ and ApoE^−/−^NPRC^−/−^ mice by ELISA (*n* = 5 per group). **j** Representative DHE staining of ROS in the aortic root from ApoE^−/−^ and ApoE^−/−^NPRC^−/−^ mice (scale = 50 μm). **k** Quantification of mean fluorescence intensity of ROS in the aortic root from ApoE^−/−^ and ApoE^−/−^NPRC^−/−^ mice. **l** Quantification of oxLDL level in serum and aorta from NPRC^ecWT^ and NPRC^ecKO^ mice by ELISA (*n* = 5 per group). **m** Representative DHE staining of ROS in the aortic root from NPRC^ecWT^ and NPRC^ecKO^ mice (scale = 50 μm). **n** Quantification of mean fluorescence intensity of ROS in the aortic root from NPRC^ecWT^ and NPRC^ecKO^ mice. Normal distributions were tested by Shapiro–Wilk method. Unpaired two-tailed Student’s t tests were applied in (**a**), (**c**), (**e**), (**g**), (**h**), (**i**), (**l**), and (**n**)

First, we compared the NPRC expression level in HAECs, VSMCs, and macrophages in vitro by qPCR, and found that NPRC expression was lower in VSMCs than in HAECs, and extremely low in macrophages (Supplementary Fig. 3e), suggesting that NPRC deletion in endothelial cells may be sufficient to achieve the beneficial effect. In addition, we compared the NPRC expression level between atherosclerotic lesions and lesion-free wall of the aortic root by inmmunofluoresence, and found that NPRC expression was upregulated primarily in endothelial cells and much less in smooth muscle cells (Supplementary Fig. 3f–g). For this reason, we crossed Tek-Cre mice with NPRC^flox/flox^ mice to derive NPRC^ecKO^ mice in the third part of in vivo experiments. NPRC^ecKO^ mice and their littermate NPRC^ecWT^ mice were fed with Western diet for 16 weeks after receiving an injection of PCSK9 AAV through tail veins. At the end of the experiment, no significant difference in the serum levels of TG, TC, LDL-C, HDL-C, and VLDL-C between NPRC^ecKO^ and littermate NPRC^ecWT^ mice (Supplementary Fig. 3h) was found. Deficient NPRC expression in aortas of NPRC^ecKO^ mice was confirmed by immunofluorescence (Supplementary Fig. 3i, j). Compared with NPRC^ecWT^ mice, NPRC^ecKO^ mice displayed 27% reduction of atherosclerotic lesion area (*P* < 0.01) as demonstrated by Oil-red O staining of the en face aorta (Fig. 3a). Similarly, the cross-sectional area of atherosclerotic lesions in the aortic root was decreased by 35% (*P* < 0.001) as displayed by H-&E staining in NPRC^ecKO^ mice versus NPRC^ecWT^ mice (Fig. 3b, c). These results indicated that endothelial cell-specific NPRC deletion attenuated atherosclerotic lesions, albeit to a less degree than systemic NPRC deletion, possibly due to the fact that NPRC is also expressed in VSMCs (Supplementary Fig. 3e–g). Furthermore, the relative content of MOMA2^+^-macrophage/monocytes and Oil-red O positive staining area in these lesions were reduced by 19% (*P* < 0.01) and 13% (*P* < 0.05), respectively, in NPRC^ecKO^ mice relative to the littermate NPRC^ecWT^ mice (Fig. 3b, c), suggesting that the extent of inflammation and the lipid deposition in atherosclerotic lesions were substantially mitigated by endothelial cell-specific NPRC deletion. In contrast, the relative content of smooth muscle cells as measured by alpha-smooth muscle actin positive staining area, and that of collagen as measured by Sirius-red positive stained area in atherosclerotic lesions were increased by 50% (*P* < 0.001) and 53% (*P* < 0.001), respectively, in NPRC^ecKO^ mice in comparison with the littermate NPRC^ecWT^ mice (Fig. 3b, c). Consequently, the vulnerability index of atherosclerotic lesions was reduced by 60% (*P* < 0.001) in NPRC^ecKO^ mice versus the littermate NPRC^ecWT^ mice (Fig. 3c). Consistently, the expression levels of pro-inflammatory cytokines including TNFα, MCP1, IL-6, ICAM1, and VCAM1 were significantly lower in the aortic tissues of NPRC^ecKO^ mice than NPRC^ecWT^ mice, as demonstrated by immunohistochemical staining (Fig. 3d, e). These data indicated that endothelial cell-specific NPRC deletion attenuated local and systemic inflammation and enhanced the stability of atherosclerotic lesions.

### NPRC defection alleviates oxidative stress in the aortic tissues and HAECs by rescuing eNOS expression

**Fig. 4:**
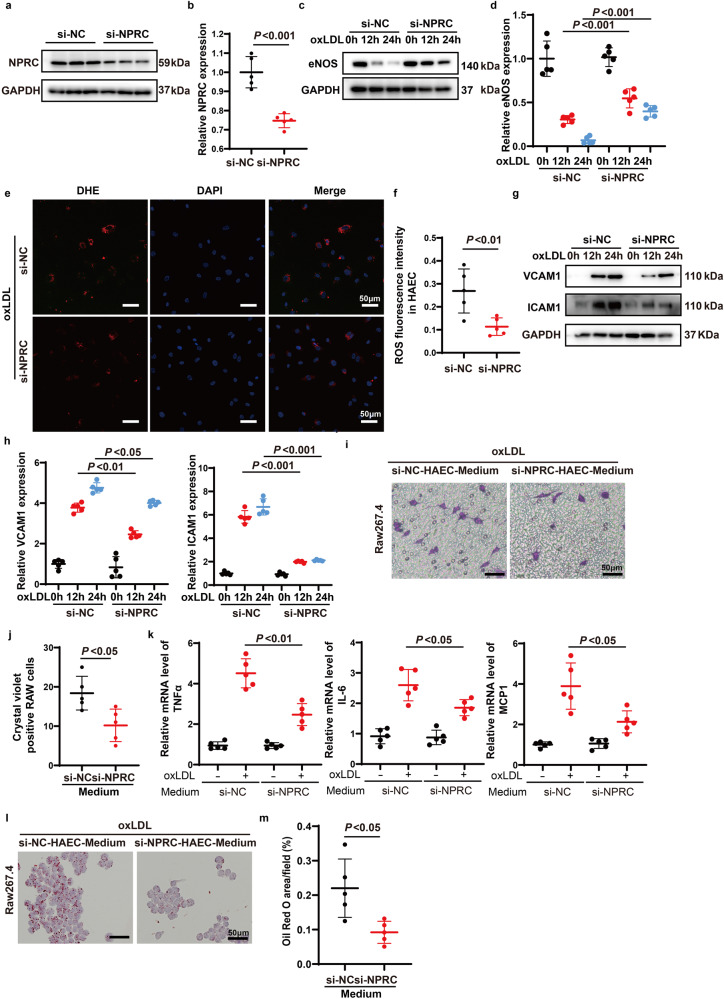
Loss of NPRC increased eNOS expression and alleviated endothelial oxidative stress and inflammation. **a** Representative Western blot images of NPRC expression in si-NC (Negative Control) and si-NPRC (NPRC-knockdown) HAECs. **b** Quantification of NPRC expression in si-NC and si-NPRC HAECs (*n* = 5 per group). **c** Representative Western blot images of eNOS expression in oxLDL-stimulated si-NC and si-NPRC HAECs. **d** Quantification of eNOS expression in oxLDL-stimulated si-NC and si-NPRC HAECs (*n* = 5 per group). **e** Representative DHE staining of ROS in oxLDL-stimulated si-NC and si-NPRC HAECs. **f** Quantification of mean fluorescence intensity of ROS in oxLDL-stimulated si-NC and si-NPRC HAECs (*n* = 5 per group). **g** Representative Western blot images of VCAM1 and ICAM1 expression in oxLDL-stimulated si-NC and si-NPRC HAECs. **h** Quantification of VCAM1 and ICAM1 expression in oxLDL-stimulated si-NC and si-NPRC HAECs (*n* = 5 per group). **i** Representative images of Raw 264.7 migration from oxLDL-stimulated si-NC and si-NPRC HAECs (scale bar = 50 μm). **j** Quantification of crystal violet positive cells in Raw 264.7 stimulated by medium from si-NC and si-NPRC HAECs (*n* = 5 per group). **k** Quantification of mRNA expression levels of MCP1, TNFα, and IL-6 in si-NC and si-NPRC HAECs after oxLDL stimulation at indicated time points (*n* = 5 per group). **l** Representative images of phagocytosis of Raw 264.7 stimulated by medium from si-NC and si-NPRC HAECs after oxLDL stimulation (scale bar = 50 μm). **m** Quantification of relative positive staining area of Oil Red O (*n* = 5 per group). Normal distributions were tested by Shapiro–Wilk method. Unpaired two-tailed Student’s t tests were applied in (**b**), (**f**), (**j**), and (**m**). Two-way ANOVA was utilized in (**d**), (**h**), and (**j**)

As nitric oxide (NO), synthesized and released by vascular endothelial cells, activates the PKG signaling pathway in VSMCs to keep vessel dilated, and endothelial NO synthase (eNOS) is the dominant enzyme to catalyze the formation of NO in endothelial cells, eNOS plays a key role in maintaining endothelial function and vascular homeostasis.^29^ In the aorta of ApoE^−/−^NPRC^−/−^ mice in the second part of in vivo experiments, we found enhanced expression of eNOS compared to ApoE^−/−^ mice by Western blot and qPCR (Fig. 3f–h). In addition, the expression of p-eNOS in the aortic root was upregulated in ApoE^−/−^NPRC^−/−^ mice versus ApoE^−/−^ mice, and in NPRC^ecKO^ mice versus NPRC^ecWT^ mice by inmmunofluoesence (Supplementary Fig. 4a–d). As endothelium-derived NO also acts as an antioxidant to inhibit LDL oxidation and peroxidases, we measured the oxidized LDL level in serum and aortic tissues and ROS level in the aortic root of mice. We found that the oxidized LDL level in serum and aortic tissues and ROS level in the aortic root assayed by DHE staining were reduced in ApoE^−/−^NPRC^−/−^ mice versus ApoE^−/−^ mice (Fig. 3i–k). These results were further verified by the evidence that the oxidized LDL level in the aortic tissues and ROS level in the aortic root were also attenuated in NPRC^ecKO^ mice compared with the littermate NPRC^ecWT^ mice in the third part of in vivo experiments (Fig. 3l–n). Similarly, oxLDL stimulation time-dependently downregulated expression of eNOS in HAECs in vitro, whereas knockdown of NPRC rescued substantially eNOS expression in HAECs treated with oxLDL (Fig. 4a–d). Moreover, ROS production induced by oxLDL in HAECs was alleviated by knockdown of NPRC (Fig. 4e, f). These results suggested that NPRC deletion protected endothelial cells from oxidative stress by rescuing eNOS expression.

### NPRC knockdown inhibits macrophage migration, cytokine expression, and phagocytosis via effects on HAECs in vitro

Previous studies reported that endothelial cells are activated during the early phase of atherosclerosis, with resultant secretion of adhesive molecules and chemokines to recruit macrophages into the vascular wall. Thus, we examined whether knockdown of endothelial NPRC affects expression of adhesive molecules and chemokines in HAECs, and the results showed that expression of pro-inflammatory adhesive molecules, such as ICAM1 and VCAM1, was upregulated time-dependently after oxLDL stimulation, whereas this trend was markedly attenuated by NPRC knockdown (Fig. 4g, h) in HAECs. We found that Raw 264.7 cells exhibited less migration stimulated by the medium of NPRC-knockdown HAECs than that by the medium of control HAECs (Fig. 4i, j). In addition, qPCR demonstrated increased expression of TNFα, IL-6, and MCP-1 in peritoneal macrophages stimulated by the medium from control HAECs after oxLDL treatment. In contrast, expression of these cytokines was downregulated in peritoneal macrophages stimulated by the medium from NPRC-knockdown HAECs after oxLDL treatment (Fig. 4k). Finally, macrophages stimulated by the medium from NPRC-knockdown HAECs showed less phagocytosis of lipids than those stimulated by the medium from control HAECs (Fig. 4l, m). Taken together, these results demonstrated that NPRC knockdown inhibited pro-inflammatory cytokine expression in HAECs, which in turn suppressed macrophage migration, cytokine expression, and phagocytosis.

### NPRC knockdown attenuates apoptosis of HAECs via enhancing AKT1 phosphorylation in vitro

**Fig. 5:**
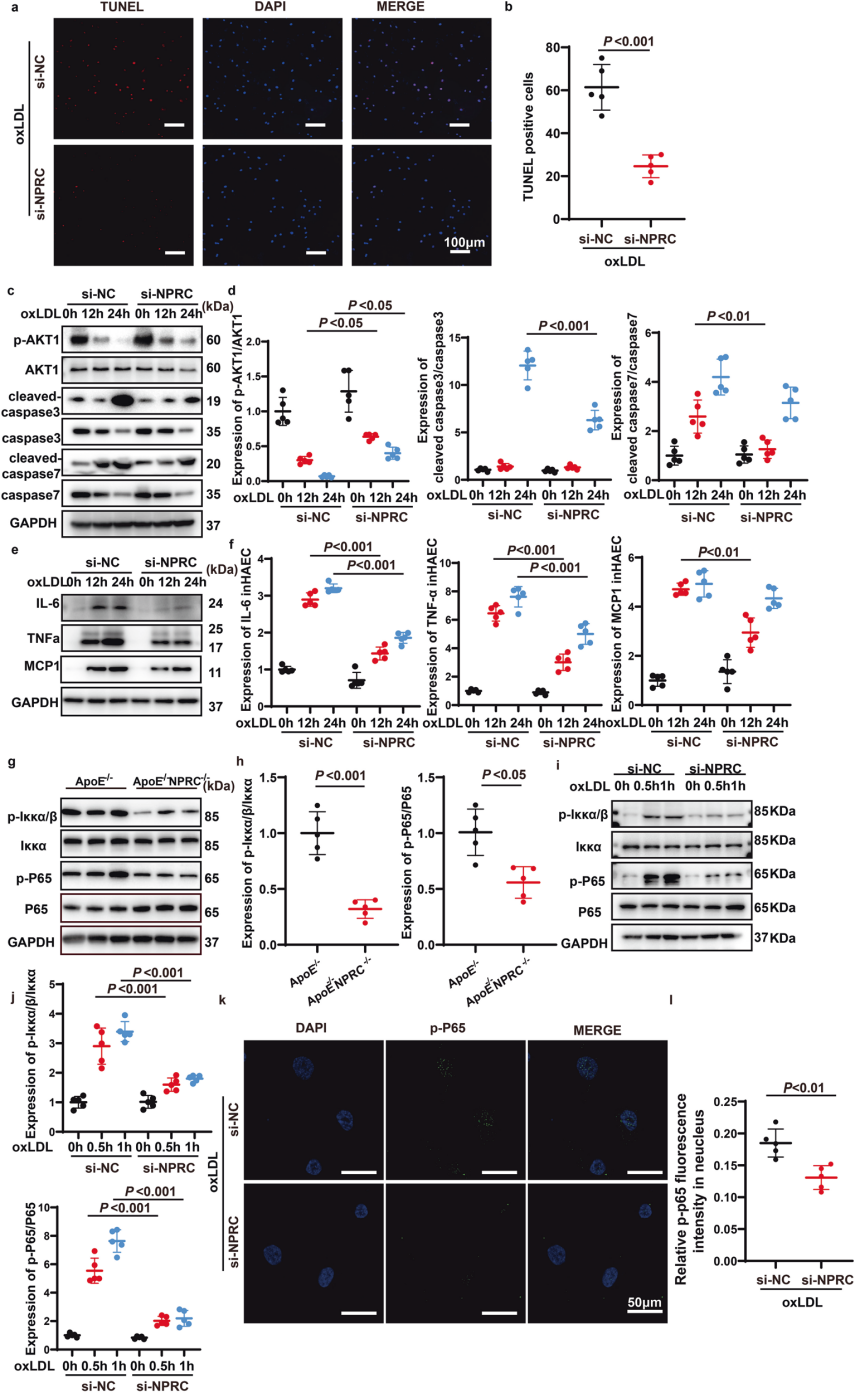
Loss of NPRC increased AKT1 phosphorylation, mitigated oxidative stress, inflammation, and apoptosis. **a** Representative images of TUNEL assay in si-NC and si-NPRC HAECs stimulated by oxLDL (scale bar = 100 μm). **b** Quantification of number of TUNEL-positive cells in si-NC and si-NPRC HAECs stimulated by oxLDL (*n* = 5 per group). **c** Representative Western blot images of p-AKT1, AKT1, Cleaved-caspase 3, Caspase 3, Cleaved-caspase 7, and Caspase 7 expression in oxLDL-stimulated si-NC and si-NPRC HAECs. **d** Quantification of p-AKT1, AKT1, Cleaved-caspase 3, Caspase 3, Cleaved-caspase 7, and Caspase 7 expression in oxLDL-stimulated si-NC and si-NPRC HAECs (*n* = 5 per group). **e** Representative Western blot images of IL-6, TNFα, and MCP1 in oxLDL-stimulated si-NC and si-NPRC HAECs. **f** Quantification of IL-6, TNFα, and MCP1 expression in oxLDL-stimulated si-NC and si-NPRC HAECs (*n* = 5 per group). **g** Representative Western blot images of p-Iκκα/β, Iκκα, p-P65, and P65 expression in aorta from ApoE^−/−^ and ApoE^−/−^NPRC^−/−^ mice. **h** Quantification of p-Iκκα/β/Iκκ and p-P65/P65 expression in aorta from ApoE^−/−^ and ApoE^−/−^ NPRC^−/−^ mice (*n* = 5 per group). **i** Representative Western blot images of p-Iκκα/β, Iκκα, Iκκβ, p-P65, and P65 expression in oxLDL-stimulated si-NC and si-NPRC HAECs. **j** Quantification of p-Iκκα/β, Iκκα, Iκκβ, p-P65, and P65 expression in oxLDL-stimulated si-NC and si-NPRC HAECs (*n* = 5 per group). **k** Representative immunofluorescence images of p-P65 in si-NC and si-NPRC HAECs stimulated by oxLDL (scale bar = 50 μm). **l** Quantification of p-P65 immunofluorescent intensity in nuclei of si-NC and si-NPRC HAECs stimulated by oxLDL (*n* = 5 per group). Normal distributions were tested by Shapiro–Wilk method. Unpaired two-tailed Student’s t tests were applied in (**b**), (**g**), and (**l**). Two-way ANOVA was used in (**d**), (**f**), and (**j**)

In the process of atherosclerosis, increased apoptosis of vascular endothelial cells may destruct endothelial integrity and permit pro-inflammatory cells to migrate into the arterial wall.^3^ In this study, we first examined the apoptotic status of HAECs in vitro by TUNEL after stimulation of oxLDL, and found that oxLDL increased the number of apoptotic HAECs, whereas NPRC knockdown in HAECs effectively attenuated apoptosis induced by oxLDL (Fig. 5a, b). In addition, we measured the expression levels of several apoptosis-related proteins, such as cleaved-caspase3 and cleaved-caspase7, which were decreased in NPRC-knockdown HAECs versus control HAECs (Fig. 5c, d). As p-AKT1 plays an important role in protecting endothelial cells from apoptosis,^30^ we further examined the phosphorylation levels of AKT1, which were found to be time-dependently declined in control HAECs after oxLDL stimulation while this trend was partially rescued in NPRC-knockdown HAECs receiving the same oxLDL treatment (Fig. 5c, d). These results demonstrated that NPRC knockdown prevented endothelial cell apoptosis induced by oxLDL via enhancing phosphorylation of AKT1.

### NPRC knockdown suppresses inflammation via downregulating NF-κB pathway in vivo and in vitro

Previous studies showed that activated NF-κB signaling pathway induces transcription of cell adhesion molecules, such as ICAM1, VCAM1, E-selectin and P-selectin, and pro-inflammatory chemokines and cytokines including MCP1, IL-6, and TNFα, in endothelial cells.^31^ In the present study, NPRC-knockdown HAECs exhibited a markedly reduction of MCP1, TNFα, and IL-6 expression displayed by Western blot (Fig. 5e, f) and qPCR (Supplementary Fig. 4e). In addition, the levels of MCP1, TNFα and IL-6 in the liquid supernatant of control HAECs were remarkably elevated after oxLDL stimulation whereas these cytokine levels in the liquid supernatant of NPRC-knockdown HAECs were much lower after oxLDL stimulation (Supplementary Fig. 4f). More importantly, we found a much lower phosphorylation level of P65 and IKKα/β in the aortic tissues from ApoE^−/−^NPRC^−/−^ mice than ApoE^−/−^ mice in the second part of in vivo experiments (Fig. 5g, h). In addition, we found a significant and time-dependent increase in the phosphorylation level of P65 and IKKα/β after oxLDL stimulation in control HAECs whereas the phosphorylation level of p65 and IKKα/β after oxLDL stimulation was significantly attenuated in NPRC-knockdown HAECs (Fig. 5i, j). We also found less translocation of p-P65 protein into nucleus in NPRC-knockdown HAECs than in control HAECs (Fig. 5k, l). These results suggested that knockdown of NPRC in HAECs alleviated inflammation via inhibiting pro-inflammatory NF-κB signaling pathway.

### NPRC deletion activates cAMP/PKA and cGMP/PKG pathways in vivo

**Fig. 6:**
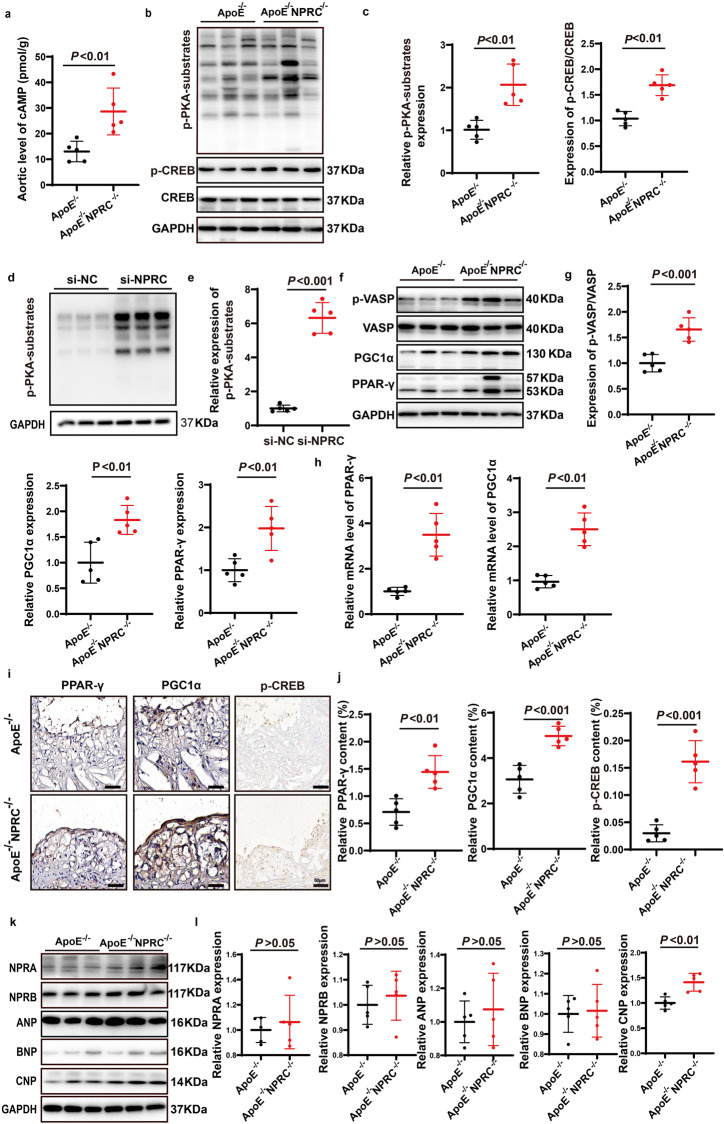
Loss of NPRC activated cAMP/PKA and cGMP/PKG pathways in vivo and in vitro. **a** Quantification of cAMP level in aorta from ApoE^−/−^ and ApoE^−/−^ NPRC^−/−^ mice (*n* = 5 per group). **b** Representative Western blot images of p-PKA-substrate, p-CREB and CREB expression in aorta from ApoE^−/−^ and ApoE^−/−^NPRC^−/−^ mice. **c** Quantification of p-PKA-substrate and p-CREB/CREB expression in aorta from ApoE^−/−^ and ApoE^−/−^NPRC^−/−^ mice (*n* = 5 per group). **d** Representative Western blot images of p-PKA-substrate expression in si-NC and si-NPRC HAECs. **e** Quantification of p-PKA-substrate expression in si-NC and si-NPRC HAECs (*n* = 5 per group). **f** Representative Western blot images of p-VASP, VASP, PGC1α, and PPAR-γ expression in aorta from ApoE^−/−^ and ApoE^−/−^NPRC^−/−^ mice. **g** Quantification of p-VASP/VASP, PGC1α, and PPAR-γ expression in aorta from ApoE^−/−^ and ApoE^−/−^NPRC^−/−^ mice (*n* = 5 per group). **h** Quantification of mRNA levels of PGC1α and PPAR-γ in aorta of ApoE^−/−^ and ApoE^−/−^ NPRC^−/−^ mice (*n* = 5 per group). **i** Representative images of immunohistochemical staining of PGC1α, PPAR-γ, and p-CREB in aorta of ApoE^−/−^ mice and ApoE^−/−^NPRC^−/−^ mice (scale bar = 50 μm). **j** Quantification of relative positive staining area of PGC1α, PPAR-γ, and p-CREB (*n* = 5 per group). **k** Representative Western blot images of NPRA, NPRB, ANP, BNP, and CNP expression in aorta from ApoE^−/−^ and ApoE^−/−^NPRC^−/−^ mice. **l** Quantification of NPRA, NPRB, ANP, BNP, and CNP expression in aorta from ApoE^−/−^ and ApoE^−/−^NPRC^−/−^ mice (*n* = 5 per group). Normal distributions were tested by Shapiro–Wilk method. Unpaired two-tailed Student’s t tests were applied in (**a**), (**c**), (**e**), (**g**), (**h**), (**j**), and (**l**)

In addition to the role as a clearance receptor for ANP, BNP and CNP, NPRC has been found to inhibit guanine nucleotide regulatory protein (Gi) via its catalytic activity, which may decrease cyclic adenosine monophosphate (cAMP) levels. To explore the molecular mechanism of attenuated inflammation in ApoE^−/−^NPRC^−/−^ mice, we examined the changes of cAMP/PKA signaling pathway in the second part of in vivo experiments. First, we measured the expression level of cAMP by chemiluminescent assay, and the phosphorylation level of phospho-PKA substrate (RRXS*/T*) and phosphorylated cAMP response element binding protein (CREB) in the mouse aorta by Western blot, and found that cAMP level and phosphorylation level of RRXS*/T* and CREB in aortic tissues were significantly increased in ApoE^−/−^NPRC^−/−^ mice compared with ApoE^−/−^ mice, suggesting a considerable activation of cAMP/PKA/p-CREB pathway in the former group (Fig. 6a–c). We then examined the relation between NPRC knockdown and activation of PKA in endothelial cells and found that phospho-PKA substrates (RRXS*/T*) increased markedly in NPRC-knockdown HAECs relative to control HAECs, as shown by Western blot (Fig. 6d, e), which was similar to PKA activation in vivo. Thereafter, we examined the expression level of downstream proteins of PKA, such as PPARγ and PGC1α, and found that expression of these proteins was upregulated in ApoE^−/−^NPRC^−/−^ mice versus ApoE^−/−^ mice as demonstrated by Western blot (Fig. 6f, g) and qPCR (Fig. 6h). Similarly, the expression level of PPAR-γ and PGC1α was higher in the aortic tissues of ApoE^−/−^NPRC^−/−^ mice than ApoE^−/−^ mice as shown by immunohistochemical staining (Fig. 6i, j). In addition, we found that the phosphorylation level of vasodilator-stimulated phosphoprotein (VASP) was upregulated in ApoE^−/−^NPRC^−/−^ mice relative to ApoE^−/−^ mice, suggesting that cGMP/PKG/p-VASP pathway was activated as well (Fig. 6f, g), possibly due to a defect of clearance for natriuretic peptides, especially CNP, in ApoE^−/−^NPRC^−/−^ mice. To test this hypothesis, we measured the serum levels and protein expression levels of ANP, BNP, and CNP in the two groups of mice. The serum levels of ANP and CNP were substantially higher in ApoE^−/−^NPRC^−/−^ mice than ApoE^−/−^ mice (Supplementary Fig. 5), while no difference was detected in the serum levels of BNP between the two groups of mice, likely due to the fact that the physiological level of BNP is much lower than ANP and CNP because secretion of BNP is mainly driven by atrial dilatation. As expected, the protein expression of NPRA and NPRB in the aortic tissues was similar between the two groups of mice (Fig. 6k, l). Similarly, the expression levels of ANP and BNP in the aortic tissues showed no difference between ApoE^−/−^NPRC^−/−^ and ApoE^−/−^ mice while CNP exhibited a much higher expression level in the former than the latter group, probably because ANP and BNP are secreted by cardiomyocytes while CNP is secreted by vascular endothelial cells (Fig. 6k, l). Taken together, these results indicated that NPRC deletion activated both cAMP/PKA and cGMP/PKG pathways in ApoE^−/−^NPRC^−/−^ mice.

### NPRC overexpression in endothelium aggravates atherosclerotic lesions in vivo

**Fig. 7:**
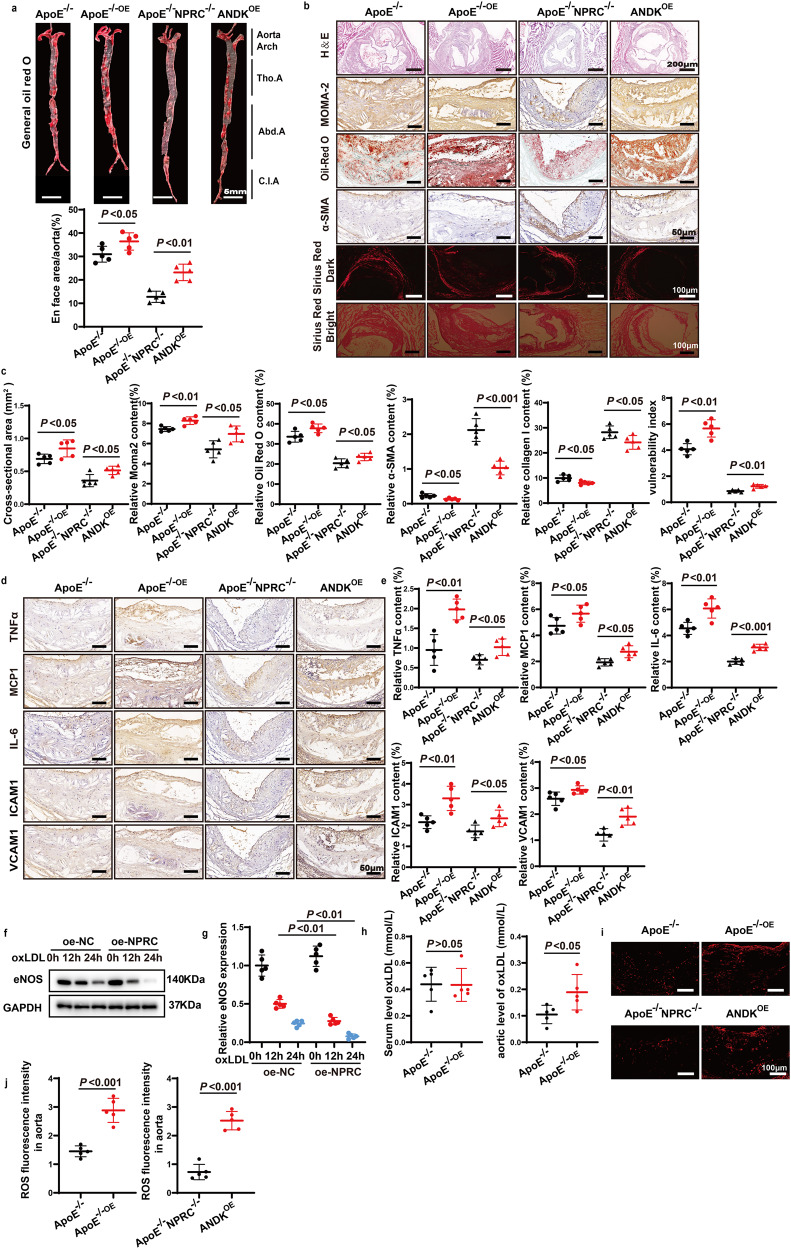
Comparison of atherosclerotic lesions, inflammatory cytokine expression, and oxidative stress between ApoE^−/−^ and ApoE^−/−OE^ mice and between ApoE^−/−^NPRC^−/−^ and ANDK^OE^ mice. **a** Representative images of Oil Red O staining of en face aorta (upper panel) and quantification of Oil red O positive staining area (lower panel) in ApoE^−/−^, ApoE^−/−OE^, ApoE^−/−^NPRC^−/−^, and ANDK^OE^ mice (*n* = 5 per group) (scale bar = 5 mm). **b** Representative images of HE staining (scale bar = 200 μm), Oil Red O staining (scale bar = 50 μm), Sirius red staining (scale bar = 100 μm), and immunohistochemical staining for Moma2 (scale bar = 50 μm) and α-SMA (scale bar = 50 μm) in the aortic tissues of ApoE^−/−^, ApoE^−/−OE^, ApoE^−/−^NPRC^−/−^ and ANDK^OE^ mice. **c** Quantification of cross-sectional and en face aortic plaque area, and Moma2, α-SMA, and collagen I positive staining area in ApoE^−/−^, ApoE^−/−OE^, ApoE^−/−^NPRC^−/−^, and ANDK^OE^ mice (*n* = 5 per group). **d** Representative images of immunohistochemical staining for TNFα, MCP1, IL-6, ICAM1, and VCAM1 in atherosclerotic lesions of ApoE^−/−^, ApoE^−/−OE^, ApoE^−/−^NPRC^−/−^, and ANDK^OE^ mice (scale = 50 μm). **e** Quantification of TNFα, MCP1, IL-6, ICAM1, and VCAM1 positive staining area in atherosclerotic lesions of ApoE^−/−^, ApoE^−/−OE^, ApoE^−/−^NPRC^−/−^, and ANDK^OE^ mice (*n* = 5 per group). **f** Representative Western blot images of eNOS expression in oxLDL-stimulated oe-NC and oe-NPRC HAECs. **g** Quantification of eNOS expression in oxLDL-stimulated oe-NC and oe-NPRC HAECs (*n* = 5 per group). **h** Quantification of oxLDL level in serum and aorta from ApoE^−/−^ mice and ApoE^−/−oe^ mice by ELISA (*n* = 5 per group). **i** Representative images of DHE staining of ROS in the aortic root from ApoE^−/−^, ApoE^−/−OE^, ApoE^−/−^NPRC^−/−^, and ANDK^OE^ mice (scale = 50 μm). **j** Quantification of mean fluorescence intensity of ROS in the aortic root from ApoE^−/−^, ApoE^−/−OE^, ApoE^−/−^NPRC^−/−^, and ANDK^OE^ mice. Normal distributions were tested by Shapiro–Wilk method. One-way ANOVA tests were applied in (**a**), (**c**), (**e**). Unpaired two-tailed Student’s t tests were applied in (**h**) and (**j**). Two-way ANOVA was used in (**f**)

To further verify the role of NPRC in atherosclerotic lesion formation, we overexpressed NPRC gene in vascular endothelial cells of mice by injecting AAV9 containing ICAM-2 promotor-NPRC-EGFP-3flag-WPRE-SV40_polyA into ApoE^−/−^ or ApoE^−/−^NPRC^−/−^ mice to generate ApoE^−/−OE^ or ANDK (ApoE and NPRC Double Knockout)^OE^ mice, and injecting AAV9 containing ICAM-2 promotor-EGFP-3flag-WPRE-SV40_polyA into ApoE^−/−^ or ApoE^−/−^NPRC^−/−^ mice to generate control mice in the fourth part of in vivo experiments. Enhanced NPRC expression in aortas of ApoE^−/−OE^ and ANDK^OE^ mice was confirmed by immunofluorescence (Supplementary Fig. 6a, b). There was no significant difference in the serum levels of TG, TC, LDL-C, HDL-C, and VLDL-C between ApoE^−/−OE^ and littermate ApoE^−/−^ mice or between ApoE^−/−^NPRC^−/−^ mice and littermate ANDK^OE^ mice (Supplementary Fig. 6c). Endothelial overexpression of NPRC resulted in an increase of 18% (*P* < 0.05) in atherosclerotic lesion area as demonstrated by Oil-red O staining of the en face aorta versus that of ApoE^−/−^ mice (Fig. 7a). The cross-sectional area of atherosclerotic lesions in the aortic root increased by 23% (*P* < 0.05) as displayed by H&E staining in ApoE^−/−OE^ mice versus ApoE^−/−^ mice (Fig. 7b, c). More strikingly, compared with ApoE^−/−^NPRC^−/−^ mice, the en face atherosclerotic lesion area was increased by 82% (*P* < 0.01) in ANDK^OE^ mice (Fig. 7b, c). Similarly, the cross-sectional area of atherosclerotic lesions in the aortic root increased by 44% (*P* < 0.05) as displayed by H&E staining in ANDK^OE^ mice versus ApoE^−/−^NPRC^−/−^ mice (Fig. 7b, c). These results indicated that NPRC overexpression in vascular endothelium aggravated atherosclerotic lesions on the background of ApoE^−/−^ or ApoE^−/−^NPRC^−/−^.

Next, we analyzed the cellular composition of the atherosclerotic lesions by immunostaining of the aortic root. The relative content of MOMA2^+^-macrophage/monocytes and Oil-red O positive staining area in these lesions increased by 11% (*P* < 0.01) and 12% (*P* < 0.05), respectively, in ApoE^−/−OE^ mice relative to the littermate ApoE^−/−^ mice, and enlarged by 28% (*P* < 0.05) and 15% (*P* < 0.05), respectively, in ANDK^OE^ mice relative to the littermate ApoE^−/−^NPRC^−/−^ mice, suggesting that the extent of inflammation and the lipid deposition in atherosclerotic lesions were substantially aggravated by NPRC overexpression (Fig. 7b, c). In contrast, the relative content of smooth muscle cells as measured by alpha-smooth muscle actin positive staining area, and that of collagen as measured by Sirius-red positive stained area in atherosclerotic lesions decreased by 42% (*P* < 0.05) and 19% (*P* < 0.05), respectively, in ApoE^−/−OE^ mice in comparison with the littermate ApoE^−/−^ mice, and declined by 52% (*P* < 0.001) and 14% (*P* < 0.05), respectively, in ANDK^OE^ mice relative to the littermate ApoE^−/−^NPRC^−/−^ mice (Fig. 7b, c). Consequently, the vulnerability index of atherosclerotic lesions was increased by 39% (*P* < 0.01) in ApoE^−/−OE^ mice versus the littermate ApoE^−/−^ mice and by 43% (*P* < 0.01) in ANDK^OE^ mice versus the littermate ApoE^−/−^NPRC^−/−^ mice (Fig. 7b, c). These results indicated that endothelial NPRC overexpression weakened the stability of the aortic atherosclerotic lesions on the background of ApoE^−/−^ or ApoE^−/−^NPRC^−/−^.

### NPRC overexpression increases pro-inflammatory cytokine expression in atherosclerotic lesions in vivo

In the fourth part of in vivo experiments, expression levels of pro-inflammatory cytokines and adhesive molecules including TNFα, MCP1, IL-6, ICAM1, and VCAM1 were upregulated in the aortic tissues of ApoE^−/−OE^ mice relative to ApoE^−/−^ mice, as demonstrated by immunohistochemical staining. Similarly, increased expression of TNFα, MCP1, IL-6, ICAM1, and VCAM1 were observed in the aortic tissues of ANDK^OE^ mice versus ApoE^−/−^NPRC^−/−^ mice (Fig. 7d, e). These data indicated that endothelial NPRC overexpression led to a remarkable aggravation of inflammation in ApoE^−/−^ mice.

### NPRC overexpression aggravates oxidative stress in vivo and in vitro by downregulating eNOS expression

To further elaborate the mechanism of NPRC in regulating the function of endothelial cells, NPRC was overexpressed in HAECs, which was confirmed by Western blot (Supplementary Fig. 7a, b). In accordance with aforementioned results that NPRC deletion rescued the downregulated expression of eNOS in vivo and vitro, eNOS expression was reduced in HAECs after oxLDL treatment which was further declined in NPRC overexpressed HAECs (Fig. 7f, g). The decreased eNOS expression resulted in elevated oxLDL and ROS levels in the aortic tissues in NPRC- overexpressed mice relative to control mice (Fig. 7h–j). Similarly, ROS level was increased in NPRC-overexpressed HAECs assayed by DHE staining (Supplementary Fig. 7c, d). These results suggested that NPRC overexpression aggravated oxidative stress in vivo and vitro induced by pro-atherogenic stimulation.

### NPRC overexpression promotes pro-inflammatory cytokine expression, macrophage migration and phagocytosis in vitro

**Fig. 8:**
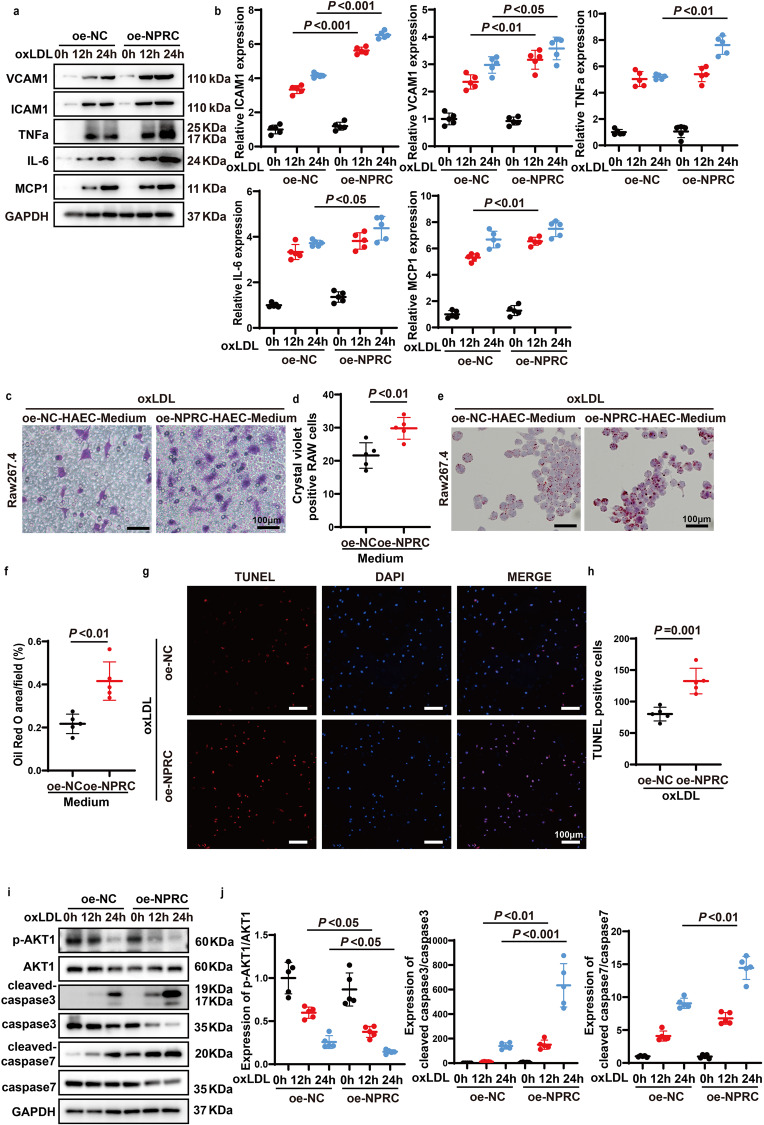
Overexpression of NPRC increased inflammation and apoptosis and aggravated migration and phagocytosis of macrophages. **a** Representative Western blot images of ICAM1, VCAM1, TNFα, MCP1, and IL-6 expression in oxLDL-stimulated oe-NC and oe-NPRC HAECs. **b** Quantification of ICAM1, VCAM1, TNFα, MCP1, and IL-6 expression in oxLDL-stimulated oe-NC and oe-NPRC HAECs (*n* = 5 per group). **c** Representative images of Raw 264.7 migration in oxLDL-stimulated oe-NC and oe-NPRC HAECs (scale bar = 100 μm). **d** Quantification of crystal violet positive cells in Raw 264.7 stimulated by medium from si-NC and si-NPRC HAECs (*n* = 5 per group). **e** Representative images of phagocytosis of Raw 264.7 from si-NC and oe-NPRC HAECs after oxLDL stimulation (scale bar = 100 μm). **f** Quantification of relative positive staining area of Oil Red O in (**e**) (*n* = 5 per group). **g** Representative images of TUNEL assay in oxLDL-stimulated oe-NC and oe-NPRC HAECs (scale bar = 100 μm). **h** Quantification of TUNEL-positive cells in oxLDL-stimulated oe-NC and si-NPRC HAECs (*n* = 5 per group). **i** Representative Western blot images of p-AKT1, AKT1, Cleaved-caspase 3, Caspase 3, Cleaved-caspase 7, and Caspase 7 expression in oxLDL-stimulated oe-NC and oe-NPRC HAECs. **j** Quantification of p-AKT1/AKT1, Cleaved-caspase 3/Caspase 3 and Cleaved-caspase 7/Caspase 7 expression in oxLDL-stimulated oe-NC and oe-NPRC HAECs (*n* = 5 per group). Normal distributions were tested by Shapiro–Wilk method. Unpaired two-tailed Student’s t tests were applied in (**d**), (**f**), (**h**). Two-way ANOVA was used in (**b**) and (**j**)

The expression level of pro-inflammatory cytokines and adhesive molecules including TNFα, MCP1, IL-6, ICAM1, and VCAM1, was upregulated time-dependently in HAECs after oxLDL stimulation, while this trend was markedly enhanced by NPRC overexpression in HAECs (Fig. 8a, b). In addition, Raw 264.7 cells exhibited more extensive migration stimulated by the medium of NPRC-overexpressed HAECs than by the medium of control HAECs (Fig. 8c, d). Macrophages stimulated by the medium from NPRC-overexpressed HAECs showed more phagocytosis of lipids than those stimulated by the medium from control HAECs (Fig. 8e, f). These results demonstrated that NPRC overexpression promoted pro-inflammatory cytokine expression in HAECs, which in turn increased macrophage migration and phagocytosis.

### NPRC overexpression aggregates apoptosis of HAECs via inhibiting AKT1 phosphorylation in vitro

Compared with the empty vector group, overexpression of NPRC in HAECs increased cell apoptosis induced by oxLDL (Fig. 8g, h). In addition, the expression levels of several apoptosis-related proteins, such as cleaved-caspase3 and cleaved-caspase7, were dramatically increased in NPRC-overexpressed HAECs versus empty vector-transfected HAECs (Fig. 8i, j). The phosphorylation level of AKT1, which was declined in control HAECs after oxLDL stimulation, was further decreased in NPRC-overexpressed HAECs receiving the same oxLDL treatment (Fig. 8i, j). Taken together, these results demonstrated that NPRC overexpression promoted endothelial cell apoptosis induced by oxLDL via downregulating phosphorylation of AKT1.

### NPRC overexpression increases inflammation via upregulating NF-κB pathway

**Fig. 9:**
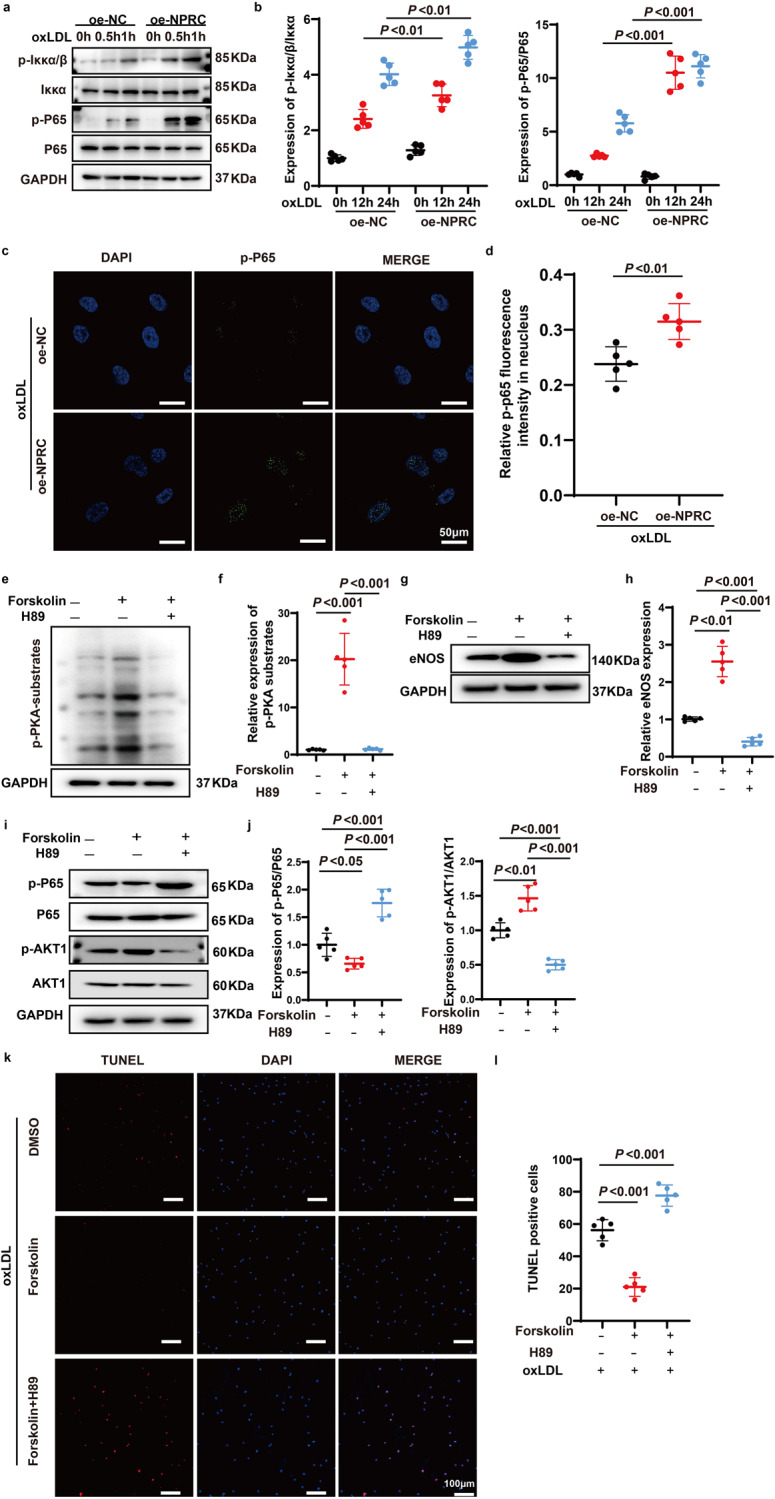
Activation of PKA pathway upregulated eNOS and p-AKT1, and inhibited p-p65 expression. **a** Representative Western blot images of p-Iκκα/β, Iκκα, Iκκβ, p-P65, and P65 expression in oxLDL-stimulated oe-NC and oe-NPRC HAECs. **b** Quantification of p-Iκκα/β, Iκκα, Iκκβ, p-P65, and P65 expression in oxLDL-stimulated oe-NC and oe-NPRC HAECs (*n* = 5 per group). **c** Representative immunofluorescence images of p-P65 in oe-NC and oe-NPRC HAECs stimulated by oxLDL (scale bar = 50 μm). **d** Quantification of p-P65 immunofluorescent intensity in nuclei of oe-NC and oe-NPRC HAECs stimulated by oxLDL (*n* = 5 per group). **e** Representative Western blot images of p-PKA-substrate expression in HAECs treated by forskolin and H89. **f** Quantification of p-PKA-substrate expression in HAECs treated by forskolin and H89 (*n* = 5 per group). **g** Representative Western blot images of eNOS expression in HAECs treated by forskolin and H89. **h** Quantification of eNOS expression in HAECs treated by forskolin and H89 (*n* = 5 per group). **i** Representative Western blot images of p-P65, P65, p-AKT1, and AKT1 expression in HAECs treated by forskolin and H89. **j** Quantification of p-P65/P65 expression in HAECs treated by forskolin and H89 (*n* = 5 per group). **k** Representative images of TUNEL assay in HAECs pretreated by forskolin and H89, and then stimulated by oxLDL (scale bar = 100 μm). **l** Quantification of TUNEL-positive cells in HAECs pretreated by forskolin and H89, and then stimulated by oxLDL (*n* = 5 per group). Normal distributions were tested by Shapiro–Wilk method. Two-way ANOVA was applied in (**b**). Unpaired two-tailed Student’s t tests were applied in (**d**). One-way ANOVAs were used in (**f**), (**h**), (**j**), and (**l**)

In the present study, NPRC-knockout in endothelial cells and NPRC-knockdown in HAECs markedly reduced the phosphorylation level of P65 and IKKα/β in vivo and in vitro, respectively. In addition, the phosphorylation level of P65 and IKKα/β was significantly increased in NPRC-overexpressed HAECs compared with that in control HAECs after oxLDL stimulation (Fig. 9a, b). Furthermore, p-P65 protein translocated into nucleus more significantly in NPRC-overexpressed HAECs than in control HAECs (Fig. 9c, d). These results suggested that overexpression of NPRC in HAECs aggravated inflammation via upregulating pro-inflammatory NF-κB signaling pathway.

### Activation of PKA signaling pathway inhibits pro-inflammatory cytokine expression and endothelial apoptosis in vitro

To understand why loss of NPRC increased the expression of eNOS in the aortic tissues and HAECs, we used forskolin, an agonist of PKA, and H89, an antagonist of PKA, to treat HAECs and found that expression of p-PKA substrates was markedly increased in HAECs treated by forskolin, which was reversed by H89 treatment in HAECs (Fig. 9e, f). Moreover, expression of eNOS was substantially enhanced in HAECs stimulated by forskolin for 24 h, which was offset by H89 treatment in HAECs (Fig. 9g, h). We then used forskolin and H89 to explore the relation between PKA pathway activation and NF-κB signaling in HAECs, and found that the phosphorylation level of P65 was significantly decreased after stimulation of forskolin but remarkably increased by H89 treatment in HAECs (Fig. 9i, j). Finally, as PKA/p-AKT1 pathway is reportedly to play a major role in modulating endothelial cell apoptosis, we further explored the relation between PKA/p-AKT1 pathway and HAECs apoptosis by using forskolin and H89. We found that treatment with forskolin increased, whereas treatment with H89 dramatically decreased, the phosphorylation level of AKT1 in HAECs (Fig. 9i, j). To verify this result, we applied forskolin and H89 to HAECs after oxLDL stimulation for 24 h, and the result showed that the number of apoptotic cells detected by TUNEL was decreased when pretreated HAECs with forskolin, but markedly increased when pretreated by H89 (Fig. 9k, l). Taken together, these data demonstrated that activation of PKA signaling pathway inhibited NF-κB signaling and endothelial apoptosis.

## Discussion

There were several important findings in the present study. First, NPRC expression was increased in atherosclerotic lesions in ApoE^−/−^ mice; second, novel mouse models of ApoE^−/−^ NPRC^−/−^ and NPRC^ecKO^ were created and NPRC deletion reduced the size and increased stability of atherosclerotic lesions; third, endothelial overexpression of NPRC in ApoE^−/−^ and ApoE^−/−^ NPRC^−/−^ mice increased the size and instability of atherosclerotic lesions; fourth, loss of NPRC lessened oxidative stress, pro-inflammatory cytokine expression and endothelial cell apoptosis, and enhanced eNOS expression, which were reversed by NPRC overexpression; fifth, NPRC knockdown inhibited macrophage migration, pro-inflammatory cytokine expression and phagocytosis via its effects on endothelial cells and overexpression of NPRC resulted in an opposite effect; finally, the mechanism underlying the beneficial effects of NPRC deletion involved activation of cAMP/PKA pathway, leading to downstream upregulated AKT1 pathway and downregulated NF-κB pathway. To the best of our knowledge, our study is the first in the literature to report the anti-atherosclerotic effects and underlying mechanisms of NPRC deletion in ApoE^−/−^ mice.

Natriuretic peptides are a family of multiple functional proteins secreted by several organs, which bind with their own receptors to play physiological roles.^32^ In the past, NPRC was deemed as a clearance receptor to degrade natriuretic peptides including ANP, BNP and CNP.^10^ However, recent studies have revealed that NPRC also acts as an important signaling receptor expressed abundantly in endothelial cells to regulate a series of cellular biological processes.^10,14–16^ Our previously reported genome-wide association study discovered that SNPs of NPRC gene contributed significantly to CAD susceptibility in the Chinese Han population.^5^ In the present study, we identified increased expression of NPRC in the atherosclerotic aorta of ApoE^−/−^ mice, implicating a potential role of NPRC in the pathogenesis of atherosclerosis. Although a previous study showed that systemic deletion of NPRC impaired angiogenesis and vascular remodeling in the setting of ischemia,^23^ the relationship between NPRC and atherosclerosis is unknown. Therefore, we generated ApoE^−/−^NPRC^−/−^ double knockout mice to establish a combined model of atherosclerosis and NPRC deletion, and littermate ApoE^−/−^ mice served as a control model of atherosclerosis. Our results revealed no significant difference in mortality, heart rate, mean blood pressure and diastolic blood pressure between ApoE^−/−^NPRC^−/−^ mice and their littermate ApoE^−/−^ mice, indicating that NPRC deletion had no effect on survival and hemodynamics in these mice. However, ApoE^−/−^NPRC^−/−^ mice exhibited a significant decrease in the serum level of triglycerides. which may be related to a less clearance of natriuretic peptides, ANP in particular, in adipose tissues caused by NPRC deletion. Wu et al generated mice with tissue-specific deletion of the NP clearance receptor, NPRC, in adipose tissue and found that natriuretic peptides can stimulate lipolysis in adipocytes and promote the “browning” of white adipose tissue.^19^ In contrast, the serum levels of TC, HDL-C, LDL-C, and VLDL-C showed no difference between ApoE^−/−^ mice and ApoE^−/−^NPRC^−/−^ mice, which is contradictory to our previous findings that increased lipolysis was associated with a decreased serum level of TG but an increased serum level of TC and LDL-C.^2–4^ This discrepancy may be explained by a previous finding that ANP prevented LDL receptor from degradation by reducing the expression of PCSK9 in adipose tissue,^33^ and thus the VLDL remnants derived from increased lipolysis of adipose tissue may be cleared away through upregulated expression of LDLR. Thus, the anti-atherosclerotic mechanism of NPRC deletion in ApoE^−/−^NPRC^−/−^ mice cannot be explained by its effect on lipid metabolism. In addition, we found that endothelial overexpression of NPRC increased the size and instability of atherosclerotic lesions in ApoE^−/−^ mice, which were rescued by endothelial cell-specific of NPRC deletion.

Endothelium, as the major regulator of vascular homeostasis, exerts a number of vasoprotective effects, such as promoting vasodilation and inhibition of oxidative stress and inflammatory responses.^23^ Since the function of vasodilation is dependent on NO synthesized and released by endothelial cells, which is regulated by eNOS, physiological and pathological factors that affect eNOS will have an impact on endothelial function.^29^ Along with the vasodilating effects by activating cGMP/PKG signaling pathway, NO has proven to decrease the expression of MCP1 in endothelial cells^34^ and inhibit leukocyte adhesion molecule CD11/CD18 expression in leukocytes.^35^ Importantly, endothelium-derived NO also acts as an antioxidant to inhibit LDL oxidation^36^ and peroxidases.^37^ In the current study, oxLDL and ROS levels in mouse aorta or endothelial cells were decreased by NPRC deletion but increased by NPRC overexpression. Moreover, oxLDL treatment resulted in a substantial reduction in NOS expression in HAECs, but this trend was reversed by NPRC knockdown in HAECs. This was consistent with the upregulated expression of eNOS and p-eNOS in atherosclerotic aorta from ApoE^−/−^NPRC^−/−^ mice compared with ApoE^−/−^ mice and NPRC^ecKO^ mice compared with NPRC^ecWT^ mice. Previous studies revealed that CREs of the eNOS promotor could be bound by p-CREB to increase eNOS expression in endothelial cells,^38^ suggesting that eNOS expression was upregulated by PKA/p-CREB signaling pathway, which was consistent with our results in this study. Paradoxically, both genetic deletion and overexpression of eNOS enhanced atherosclerosis in ApoE^−/−^ mice,^39,40^ which was explained by eNOS uncoupling induced by eNOS overexpression. Although the level of eNOS expression in NPRC-knockdown HAECs was higher than that in control HAECs after oxLDL treatment, it was not high enough to reach the baseline level of eNOS expression in control HAECs. Thus, it is conceivable that NPRC-knockdown may not induce eNOS uncoupling in HAECs.

Inflammatory infiltration is one of the most salient pathologic features of atherosclerosis in both animal models and human studies. In this study, ApoE^−/−^NPRC^−/−^ mice manifested with decreased expression of ICAM1, VCAM1, IL-6, MCP1, and TNFα in the aortic tissues and in the serum compared with ApoE^−/−^ mice, indicating an attenuated local and systemic inflammatory response in ApoE^−/−^NPRC^−/−^ mice. Although NPRC is expressed widely and abundantly in the major endocrine glands, lungs, and kidneys, a much higher expression level of NPRC was found in endothelial cells than in macrophages.^41^ Our results demonstrated a higher expression level of NPRC in HAECs than in macrophages, suggesting that the anti-inflammatory effect of NPRC deletion in ApoE^−/−^NPRC^−/−^ mice was mainly due to its effect on endothelial cells. To test this hypothesis, we generated a mouse model of endothelial cell-specific NPRC knockout (NPRC^ecKO^) who exhibited smaller and more stable atherosclerotic lesions than littermates NPRC^ecWT^ mice. In addition, we silenced NPRC gene in HAECs in vitro and found a lower level of expression of ICAM1, VCAM1, IL-6, MCP1, and TNFα after oxLDL treatment relative to that in control HAECs. Importantly, peritoneal macrophages exhibited attenuated migration, oxLDL phagocytosis and expression of IL-6, MCP1, and TNFα after stimulation with the medium of NPRC-knockdown HAECs in comparison with control HAECs after oxLDL stimulation, suggesting that NPRC-knockdown in HAECs inhibited excretion of inflammatory cytokines from HAECs induced by oxLDL. In contrast, NPRC overexpression in HAECs resulted in opposite effects. Previous studies found that in endothelial cells, activated NF-κB induced transcription of cell adhesion molecules, such as ICAM-1, VCAM-1, and chemokines and cytokines including MCP-1, M-CSF, GM-CSF, IL-6, IL-8, and TNFα.^42^ Our results demonstrated a lower phosphorylation level of Iκκα/β and P65 in the aortic tissues from ApoE^−/−^NPRC^−/−^ mice than from ApoE^−/−^ mice, and in oxLDL-treated NPRC knockdown HAECs than in oxLDL-treated control HAECs, indicating that loss of NPRC inhibited activation of NF-κB signaling pathway. As expected, these beneficial effects were reversed by NPRC overexpression in HAECs. Mechanistically, activated PKA pathway may phosphorylate PAK, which may negatively regulate PAK and suppress NF-κB signaling.^43^ In this study, forskolin, an agonist of PKA signaling, activated PKA signaling pathway and inhibited phosphorylation of p65 in HAECs, which suggested that loss of NPRC reduced inflammation by inhibiting NF-κB signaling pathway through activation of PKA in vivo and in vitro. Taken together, we believe that deficiency of NPRC decreased oxidative stress, which in turn resulted in an inhibition of inflammation and atherosclerosis. However, on both theoretical and practical ground, anti-inflammation should be the major target of NPRC deficiency.

As apoptosis of endothelial cells plays an important role in the development of atherosclerosis, we looked into the effect of NPRC knockdown on apoptosis of HAECs. Our results demonstrated that NPRC knockdown ameliorated apoptosis of HAECs induced by oxLDL, which were aggravated by NPRC overexpression in HAECs. Although physiological activation of NPRC by the endogenous ligand CNP is crucial for promoting cell proliferation, excessive activation of NPRC has been shown to exert antigrowth effects in vascular smooth muscle cells through inhibition of cAMP and AKT expression.^44^ AKT inhibition of endothelial cells was associated with endothelial damage and dysfunction.^45^ On the contrary, activation of AKT1 signaling was shown to decrease apoptosis of HAECs.^45^ In this study, NPRC knockdown in HAECs restored the phosphorylation level of AKT1 downregulated by oxLDL through activation of the PKA signaling, whereas NPRC overexpression in HAECs aggravated downregulation of the phosphorylation level of AKT1. Similarly, we found that inhibition of PKA by H89 significantly reduced the phosphorylation level of AKT1 in HAECs, which was consistent with a previous study showing that an increase of AKT1 signaling induced by M1 muscarinic receptor activation was abolished by PKA inhibitors.^46^

In the current study, we explored the signaling pathways underlying the anti-atherosclerotic effect of NPRC deletion and demonstrated that loss of NPRC activated PKA signaling pathway in vivo and in vitro. Interestingly, PKG pathway was activated as well in the aortic tissues from ApoE^−/−^NPRC^−/−^ mice. Previous studies reported that CNP activated PKG signaling pathway though binding to NPRB^15^ and NPRC deletion may lead to an increased serum level of CNP as revealed in this study. In addition, it has been reported that increased NO derived from upregulated eNOS may activate PKG signaling as well.^47^ By comparison, activation of PKA signaling pathway is a direct effect of NPRC deletion and thus, is a major molecular mechanism underling the anti-inflammation and anti-atherosclerosis effect of NPRC deletion in ApoE^−/−^NPRC^−/−^ mice.

There were several limitations in the present study. First, although we demonstrated that NPRC deletion activated PKA signaling pathway but whether inhibiting PKA may reverse the anti-atherosclerotic effect of NPRC deletion was not explored and further studies were needed to address this question. Second, the role of CNP in ApoE^−/−^NPRC^−/−^ mice was not examined although activated PKG signaling pathway observed in these mice may be related to the binding of increased CNP to NPRB and more experiments are required to clarify this point. Finally, the mechanism for increased NPRC expression in atherosclerotic lesions was not explored. Previous studies found a potent insulin-mediated and glucose-dependent upregulation of NPRC,^14,15^ and in the current study, we found the expression of PPAR-gamma, a key regulator of glucose and lipid metabolism, was substantially upregulated in the aortic tissue of NPRC^−/−^ApoE^−/−^ mice (Fig. 6F–J), which suggested a close relation between NPRC and metabolic disease such as AS. However, the exact mechanism remains to be investigated.

## Conclusions

**Fig. 10:**
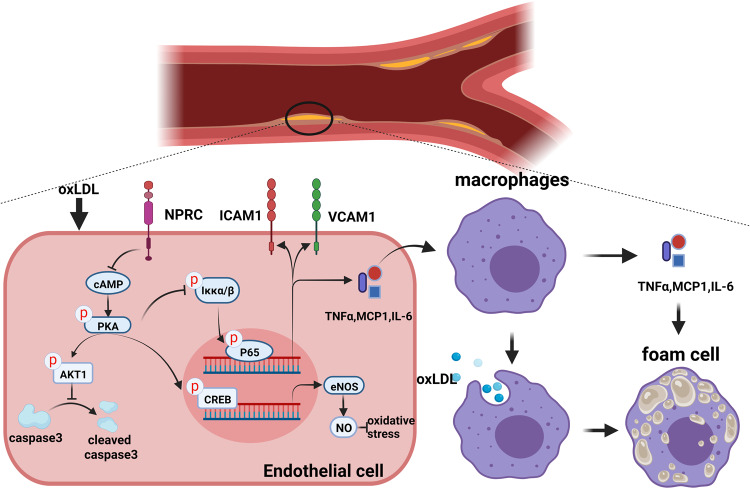
Schematic diagram showing the mechanism of NPRC-promoted atherosclerosis. Under oxLDL stimulation, NPRC inhibits cAMP/PKA signaling pathway in HAECs, which reduces eNOS expression and promotes oxidative stress, inflammatory cytokine release, and cell apoptosis, leading to foam cell formation and atherosclerosis. Diagram is generated from BioRender

NPRC expression was increased in atherosclerotic lesions and NPRC deletion reduced the size and increased stability of these lesions. Loss of NPRC attenuated oxidative stress, inflammation, and endothelial cell apoptosis and increased eNOS expression via upregulated cAMP/PKA-AKT1 pathway and downregulated NF-κB pathway (Fig. 10). Thus, targeting NPRC may provide a promising approach to prevention and treatment of atherosclerosis.

### Supplementary information

Supplementary Material

## Data Availability

All data may be obtained from the corresponding author upon reasonable request. The RNA-sequencing data generated in this study are publicly available in Gene Expression Omnibus (GEO) dataset GSE233428.

## References

[1] Virani SS (2020). Heart disease and stroke statistics-2020 update: a report from the American Heart Association. Circulation.

[2] Dong M (2013). Cold exposure promotes atherosclerotic plaque growth and instability via UCP1-dependent lipolysis. Cell Metab..

[3] Paone S, Baxter AA, Hulett MD, Poon IKH (2019). Endothelial cell apoptosis and the role of endothelial cell-derived extracellular vesicles in the progression of atherosclerosis. Cell Mol. Life Sci..

[4] Libby P (2019). Atherosclerosis. Nat. Rev. Dis. Primers.

[5] Hu Q (2016). NPR-C gene polymorphism is associated with increased susceptibility to coronary artery disease in Chinese Han population: a multicenter study. Oncotarget.

[6] Goetze JP (2020). Cardiac natriuretic peptides. Nat. Rev. Cardiol..

[7] Kuwahara K (2021). The natriuretic peptide system in heart failure: diagnostic and therapeutic implications. Pharmacol. Ther..

[8] Nakamura T, Tsujita K (2021). Current trends and future perspectives for heart failure treatment leveraging cGMP modifiers and the practical effector PKG. J. Cardiol.

[9] Bie P (2018). Natriuretic peptides and normal body fluid regulation. Compr. Physiol..

[10] Shao S (2021). Renal natriuretic peptide receptor-C deficiency attenuates NaCl cotransporter activity in angiotensin II-induced hypertension. Hypertension.

[11] Nishikimi T, Maeda N, Matsuoka H (2006). The role of natriuretic peptides in cardioprotection. Cardiovasc. Res..

[12] Suga S (1992). Endothelial production of C-type natriuretic peptide and its marked augmentation by transforming growth factor-beta. Possible existence of "vascular natriuretic peptide system". J. Clin. Invest..

[13] Leitman DC (1986). Identification of multiple binding sites for atrial natriuretic factor by affinity cross-linking in cultured endothelial cells. J. Biol. Chem.

[14] Murthy KS, Makhlouf GM (1999). Identification of the G protein-activating domain of the natriuretic peptide clearance receptor (NPR-C). J. Biol. Chem..

[15] Pagano M, Anand-Srivastava MB (2001). Cytoplasmic domain of natriuretic peptide receptor C constitutes Gi activator sequences that inhibit adenylyl cyclase activity. J. Biol. Chem..

[16] Rubattu S (2020). Epigenetic control of natriuretic peptides: implications for health and disease. Cell Mol. Life Sci..

[17] He X-l, Dukkipati A, Garcia KC (2006). Structural determinants of natriuretic peptide receptor specificity and degeneracy. J. Mol. Biol..

[18] Kanai Y (2017). Circulating osteocrin stimulates bone growth by limiting C-type natriuretic peptide clearance. J. Clin. Invest..

[19] Wu W (2017). Enhancing natriuretic peptide signaling in adipose tissue, but not in muscle, protects against diet-induced obesity and insulin resistance. Sci. Signal.

[20] Kuehnl A, Pelisek J, Pongratz J, Eckstein HH (2012). C-type natriuretic peptide and its receptors in atherosclerotic plaques of the carotid artery of clinically asymptomatic patients. Eur. J. Vasc. Endovasc. Surg..

[21] Zayed MA (2016). Natriuretic peptide receptor-C is up-regulated in the intima of advanced carotid artery atherosclerosis. J. Med. Surg. Pathol..

[22] Moyes AJ (2014). Endothelial C-type natriuretic peptide maintains vascular homeostasis. J. Clin. Invest.

[23] Bubb KJ (2019). Endothelial C-type natriuretic peptide is a critical regulator of angiogenesis and vascular remodeling. Circulation.

[24] Matsukawa N (1999). The natriuretic peptide clearance receptor locally modulates the physiological effects of the natriuretic peptide system. Proc. Natl Acad. Sci. USA.

[25] Li JJ (2012). Hepcidin destabilizes atherosclerotic plaque via overactivating macrophages after erythrophagocytosis. Arterioscler. Thromb. Vasc. Biol..

[26] Shi C (2008). Down-regulation of the forkhead transcription factor Foxp1 is required for monocyte differentiation and macrophage function. Blood.

[27] Rios FJO (2013). Uptake of oxLDL and IL-10 production by macrophages requires PAFR and CD36 recruitment into the same lipid rafts. PLoS ONE.

[28] Yi F, Zhang AY, Janscha JL, Li P-L, Zou A-P (2004). Homocysteine activates NADH/NADPH oxidase through ceramide-stimulated Rac GTPase activity in rat mesangial cells. Kidney Int..

[29] Förstermann U, Sessa WC (2012). Nitric oxide synthases: regulation and function. Eur. Heart J..

[30] Wang X (2020). Arrb2 promotes endothelial progenitor cell-mediated postischemic neovascularization. Theranostics.

[31] Jain T, Nikolopoulou EA, Xu Q, Qu A (2018). Hypoxia inducible factor as a therapeutic target for atherosclerosis. Pharmacol. Ther..

[32] McGrath MF, de Bold MLK, de Bold AJ (2005). The endocrine function of the heart. Trends Endocrinol. Metab..

[33] Bordicchia M (2019). PCSK9 is expressed in human visceral adipose tissue and regulated by insulin and cardiac natriuretic peptides. Int. J. Mol. Sci..

[34] Zeiher AM, Fisslthaler B, Schray-Utz B, Busse R (1995). Nitric oxide modulates the expression of monocyte chemoattractant protein 1 in cultured human endothelial cells. Circ. Res..

[35] Kubes P, Suzuki M, Granger DN (1991). Nitric oxide: an endogenous modulator of leukocyte adhesion. Proc. Natl Acad. Sci. USA.

[36] Li H, Förstermann U (2009). Prevention of atherosclerosis by interference with the vascular nitric oxide system. Curr. Pharm. Des..

[37] Hare JM, Stamler JS (2005). NO/redox disequilibrium in the failing heart and cardiovascular system. J. Clin. Invest..

[38] Niwano K (2006). Competitive binding of CREB and ATF2 to cAMP/ATF responsive element regulates eNOS gene expression in endothelial cells. Arterioscler. Thromb. Vasc. Biol.

[39] Kuhlencordt PJ (2001). Accelerated atherosclerosis, aortic aneurysm formation, and ischemic heart disease in apolipoprotein E/endothelial nitric oxide synthase double-knockout mice. Circulation.

[40] Ozaki M (2002). Overexpression of endothelial nitric oxide synthase accelerates atherosclerotic lesion formation in apoE-deficient mice. J. Clin. Invest..

[41] Rubattu S (2010). NPR-C: a component of the natriuretic peptide family with implications in human diseases. J. Mol. Med..

[42] Hopkins PN (2013). Molecular biology of atherosclerosis. Physiol. Rev..

[43] Funk SD (2010). Matrix-specific protein kinase A signaling regulates p21-activated kinase activation by flow in endothelial cells. Circ. Res..

[44] Sciarretta S (2013). C2238 atrial natriuretic peptide molecular variant is associated with endothelial damage and dysfunction through natriuretic peptide receptor C signaling. Circ. Res..

[45] Cheng H-W (2017). Cancer cells increase endothelial cell tube formation and survival by activating the PI3K/Akt signalling pathway. J. Exp. Clin. Cancer Res..

[46] Zhao L-X (2019). M1 muscarinic receptors regulate the phosphorylation of AMPA receptor subunit GluA1 a signaling pathway linking cAMP-PKA and PI3K-Akt. FASEB J..

[47] Riquelme JA (2016). Dexmedetomidine protects the heart against ischemia-reperfusion injury by an endothelial eNOS/NO dependent mechanism. Pharmacol. Res..

