# Correction: NPRC deletion mitigated atherosclerosis by inhibiting oxidative stress, inflammation and apoptosis in ApoE knockout mice

**DOI:** 10.1038/s41392-023-01599-x

**Published:** 2023-09-04

**Authors:** Cheng Cheng, Jie Zhang, Xiaodong Li, Fei Xue, Lei Cao, Linlin Meng, Wenhai Sui, Meng Zhang, Yuxia Zhao, Bo Xi, Xiao Yu, Feng Xu, Jianmin Yang, Yun Zhang, Cheng Zhang

**Affiliations:** 1 National Key Laboratory for Innovation and Transformation of Luobing Theory, Key Laboratory of Cardiovascular Remodeling and Function Research, Chinese Ministry of Education, Chinese National Health Commission and Chinese Academy of Medical Sciences, Department of Cardiology, Qilu Hospital of Shandong University, Jinan, China; 2 Department of Cardiology, Shengjing Hospital of China Medical University, Shenyang, Liaoning Province 110004 China; 3 Department of Traditional Chinese Medicine, Qilu Hospital, Cheeloo College of Medicine, Shandong University, Jinan, 250012 Shandong China; 4 Department of Epidemiology, School of Public Health, Cheeloo College of Medicine, Shandong University, Jinan, China; 5 Key Laboratory Experimental Teratology of the Ministry of Education, Department of Physiology, School of Basic Medical Sciences, Cheeloo College of Medicine, Shandong University, Jinan, China; 6 Department of Emergency Medicine, Chest Pain Center, Shandong Provincial Clinical Research Center for Emergency and Critical Care Medicine, Qilu Hospital, Shandong University, Jinan, China; 7 Cardiovascular Disease Research Center of Shandong First Medical University, Central Hospital Affiliated to Shandong First Medical University, Jinan, China

**Keywords:** Cardiology, Pathogenesis

Correction to: *Signal Transduction and Targeted Therapy* 10.1038/s41392-023-01560-y, published online 9 August 2023

After online publication of the article,^1^ the authors noticed one inadvertent mistake in Fig. 2b. The image of Sirus Red Dark in ApoE^−/−^ NPRC^−/−^ group was mistakenly inserted as the image of Sirus Red Bright in ApoE^−/−^ group during the final revision process. The correct figure was provided as follows.
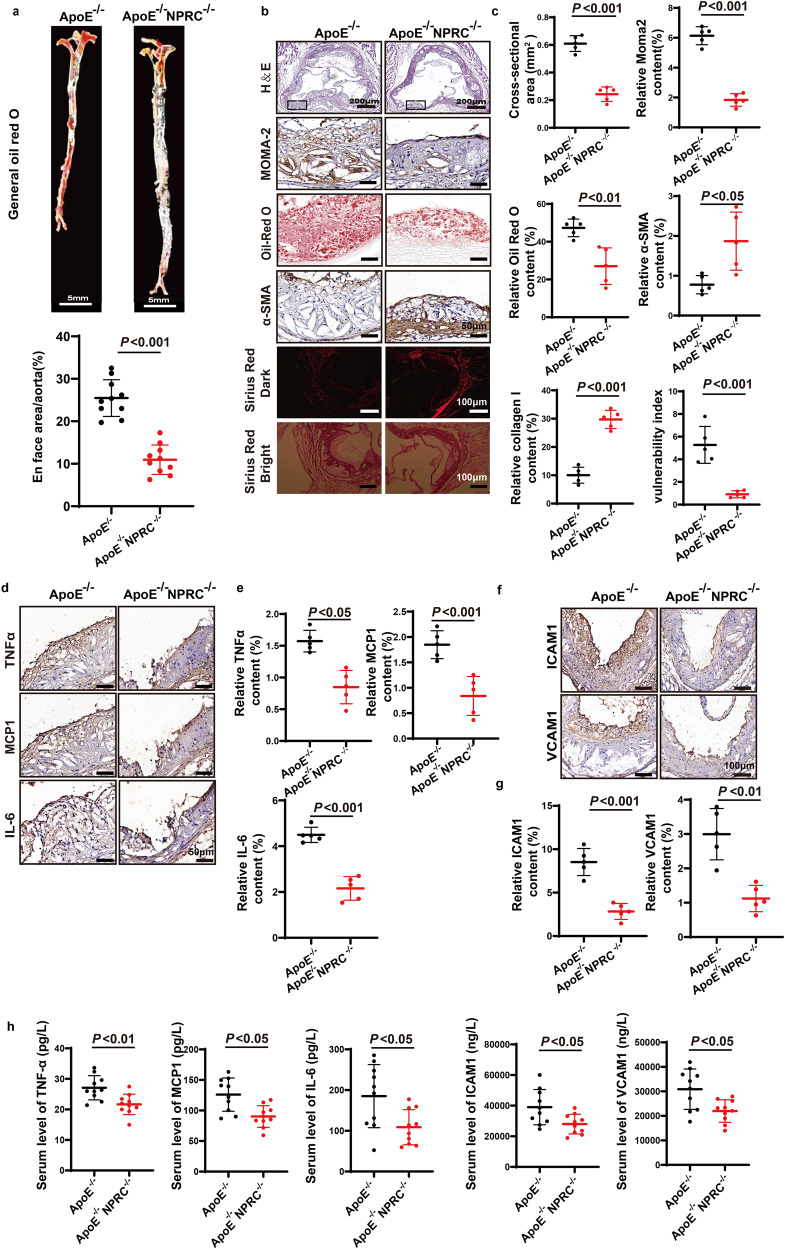


The original article has been corrected.
