# Supplementary material for: NPRC deletion mitigated atherosclerosis by inhibiting oxidative stress, inflammation and apoptosis in ApoE knockout mice
Source: Signal Transduct Target Ther. 2023 Aug 9;8:290. doi: 10.1038/s41392-023-01560-y (PMC10409771; doi:10.1038/s41392-023-01560-y)
Supplement: Supplementary file 1 — Supplementary Material [file 41392_2023_1560_MOESM1_ESM.docx]

**Supplementary Materials for**

**NPRC deletion mitigated atherosclerosis by inhibiting oxidative stress, inflammation and apoptosis in ApoE knockout mice**

Cheng Cheng^1,2^, Jie Zhang^1^, Xiaodong Li^2^, Fei Xue^1^, Lei Cao^1^, Linlin Meng^1^, Wenhai Sui^1^, Meng Zhang^1^, Yuxia Zhao^1,3^, Bo Xi^5^ , Xiao Yu^6^, Feng Xu^7^, Jianmin Yang^1^, Yun Zhang^1,4^, Cheng Zhang^1,4^

***From:***

1. National Key Laboratory for Innovation and Transformation of Luobing Theory, Key Laboratory of Cardiovascular Remodeling and Function Research, Chinese Ministry of Education, Chinese National Health Commission and Chinese Academy of Medical Sciences, Department of Cardiology, Qilu Hospital, Cheeloo College of Medicine, Shandong University, Jinan, China

2. Department of Cardiology, Shengjing Hospital of China Medical University, Shenyang, Liaoning Province, 110004, China

3. Department of Traditional Chinese Medicine, Qilu Hospital, Cheeloo College of Medicine, Shandong University, Jinan 250012, Shandong, China

4.Cardiovascular Disease Research Center of Shandong First Medical University, Central Hospital Affiliated to Shandong First Medical University, Jinan, China

5.Department of Epidemiology, School of Public Health, Cheeloo College of Medicine, Shandong University, Jinan, China

6.Key Laboratory Experimental Teratology of the Ministry of Education, Department of Physiology, School of Basic Medical Sciences, Cheeloo College of Medicine, Shandong University, Jinan, China

7.Department of Emergency Medicine, Chest Pain Center, Shandong Provincial Clinical Research Center for Emergency and Critical Care Medicine, Qilu Hospital, Shandong University, Jinan, China

***Correspondence to*:** Yun Zhang ([zhangyun@sdu.edu.cn](mailto:zhangyun@sdu.edu.cn)), or Jianmin Yang (yangjianminsdu@163.com), or Cheng Zhang ([zhangc@sdu.edu.cn](mailto:zhangc@sdu.edu.cn)).

**Cheng Cheng^1,2^, Jie Zhang^1^ and Xiaodong Li^2^ contributed equally.**

**This file includes:**

Supplementary Figures 1 to 7;

Supplementary Tables 1 to 2

Supplementary Figure 1.


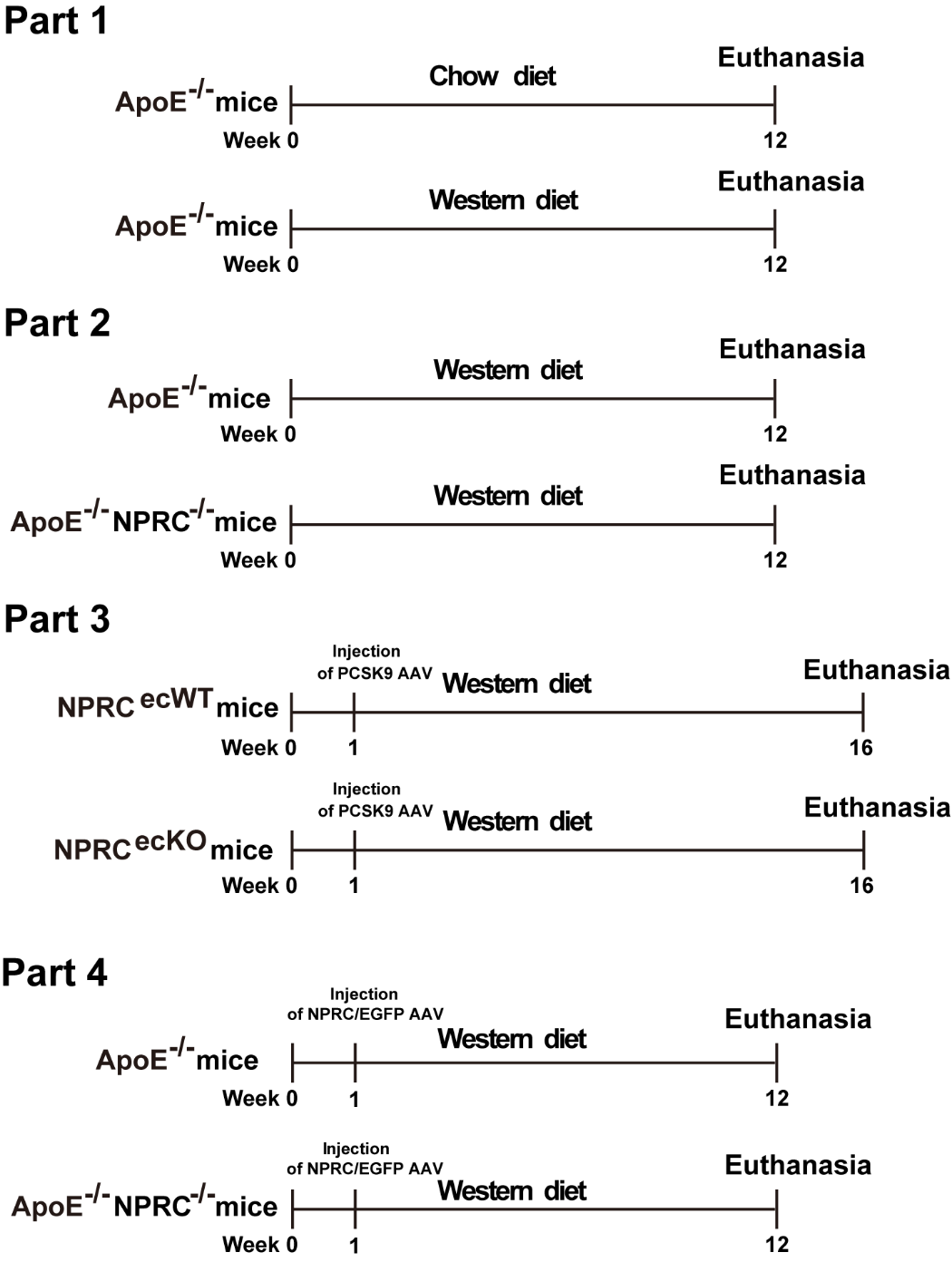


**Supplementary Figure 1.** Animal grouping and time line of *in vivo* experimental studies.

Supplementary Figure 2.


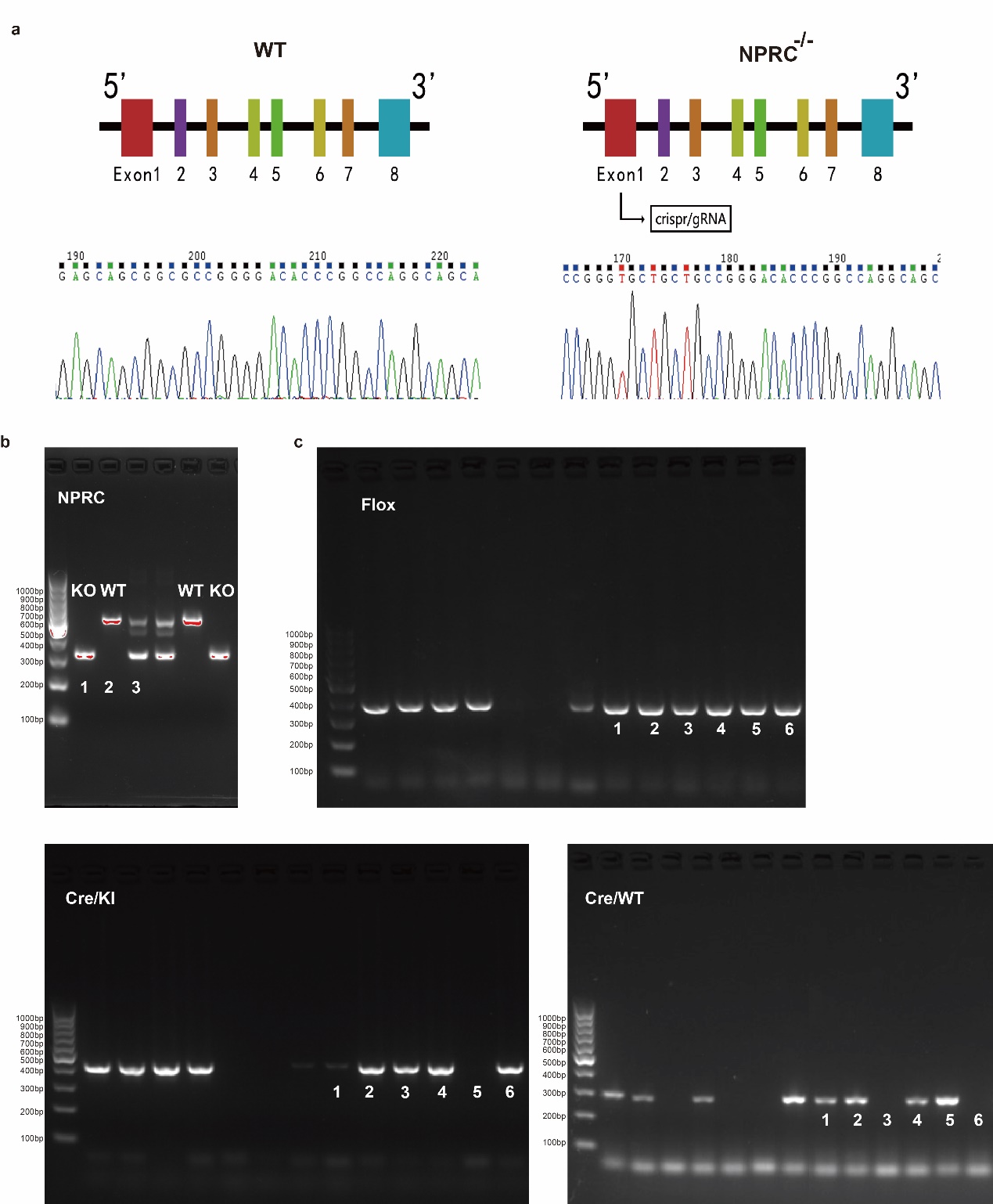


**Supplementary Figure 2.** **Generation and identification of NPRC^-/-^ and NPRC^ecKO^ mice.** **(a)** NPRC^-/-^ mice was generated using CRISPR technology. **(b)** Identification of NPRC^-/-^ mice by agarose gel electrophoresis. The genetype of number 1 to 6 mice was NPRC^-/-^, WT, NPRC^+/-^, NPRC^+/-^, WT and NPRC^-/-^, respectively. **(c)** Identification of NPRC^ecKO^ mice by agarose gel electrophoresis. The genetype of number 1 to 6 mice was Cre^WT/WT^NPRC^fl/fl^, Cre^KI/WT^NPRC^fl/fl^, Cre^KI/KI^NPRC^fl/fl^, Cre^KI/WT^NPRC^fl/fl^, Cre^WT/WT^NPRC^fl/fl^ and Cre^KI/KI^NPRC^fl/fl^, respectively. The genetype of NPRC^ecKO^ mice was Cre^KI/KI^NPRC^fl/fl^.

Supplementary Figure 3.

**
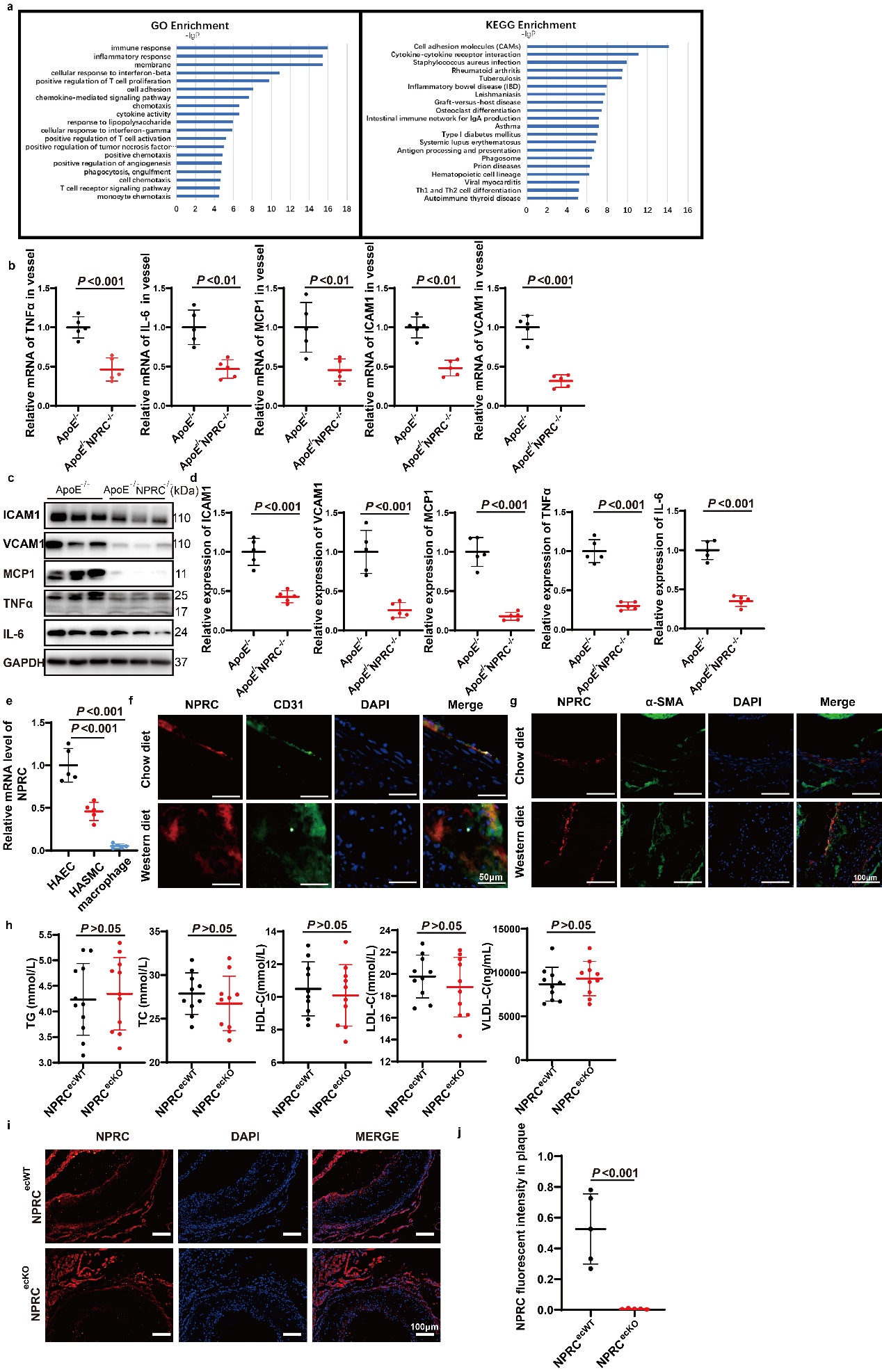
**

**Supplementary Figure 3.** **RNA high-throughput sequencing and comparison of inflammatory cytokine expression *in vivo* and the expression of NPRC in Chow Diet and Western Diet ApoE^-/-^ mice. (a)** RNA high-throughput sequencing was performed in the whole aorta of ApoE^-/-^ and ApoE^-/-^NPRC^-/-^ mice (n=5 per group). **(b)** Quantification of mRNA expression levels of TNFα, IL-6, MCP1, ICAM1 and VCAM1 in the aortic tissues form ApoE^-/-^ and ApoE^-/-^NPRC^-/-^ mice (n=5 per group). **(c)** Representative Western blot images of ICAM1, VCAM1, MCP1, TNFα and IL-6 expression in the aortic tissues from ApoE^-/-^ and ApoE^-/-^NPRC^-/-^ mice. **(d)** Quantification of ICAM1, VCAM1, MCP1, TNFα and IL-6 expression in the aortic tissues from ApoE^-/-^ and ApoE^-/-^NPRC^-/-^ mice. (n=5 per group). **(e)** Quantification of mRNA expression levels of NPRC in HAECs, HASMCs and macrophages (n=5 per group). **(f)** Representative immunofluorescence images of NPRC (red) and CD31 (green) in the aortic root from chow diet-fed and western diet-fed ApoE^-/-^ mice (scale bar=50μm). **(g)** Representative immunofluorescence images of NPRC (red) and α-SMA (green) in the aortic root from chow diet-fed and western diet-fed ApoE^-/-^ mice (scale bar=100μm). **(h)** Quantification of serum levels of TG, TC, HDL-C, LDL-C and VLDL-C in NPRC^ecWT^ and NPRC^ecKO^ mice (n=10 per group). **(i)** Representative immunofluorescence images of NPRC in the aortic root from NPRC^ecWT^ and NPRC^ecKO^ mice (scale bar=100μm). **(j)** Quantification of NPRC immunofluorescent intensity in the aortic root from NPRC^ecWT^ and NPRC^ecKO^ mice (n=5 per group). Normal distributions were tested by Shapiro-Wilk method. Unpaired two-tailed Student’s t tests were applied in (b), (d), (h) and (j). One-way ANOVA was used in (e).

Supplementary Figure 4.

**
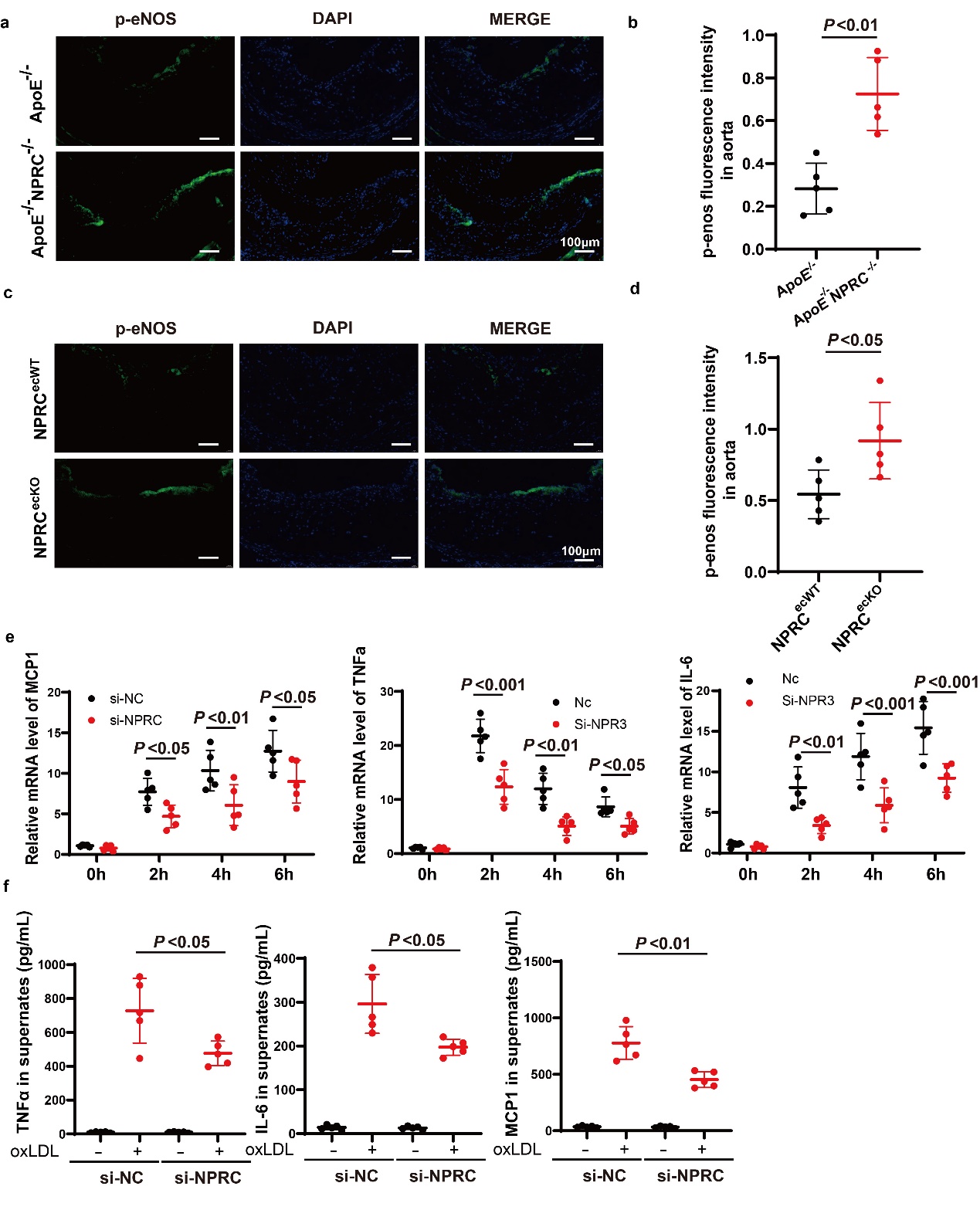
**

**Supplementary Figure 4. Comparison of p-eNOS expression between different mouse groups and inflammatory cytokine expression between different cell groups**

**(a)** Representative immunofluorescence images of p-eNOS in the aortic root from ApoE^-/-^ and ApoE^-/-^NPRC^-/-^ mice (scale bar=100μm). **(b)** Quantification of p-eNOS immunofluorescent intensity in the aortic root from ApoE^-/-^ and ApoE^-/-^NPRC^-/-^ mice. (n=5 per group). **(c)** Representative immunofluorescence images of p-eNOS in the aortic root from NPRC^ecWT^ and NPRC^ecKO^ mice (scale bar=100μm). **(d)** Quantification of p-eNOS immunofluorescent intensity in the aortic root from NPRC^ecWT^ and NPRC^ecKO^ mice. (n=5 per group). **(e)** Quantification of mRNA expression levels of TNFα, MCP1 and IL-6 in macrophages stimulated by medium from oxLDL-treated si-NC and si-NPRC HAECs (n=5 per group). **(f)** Quantification of IL-6, MCP1 and TNFα levels in supernatant from oxLDL-stimulated si-NC and si-NPRC HAECs (n=5 per group). Normal distributions were tested by Shapiro-Wilk method. Unpaired two-tailed Student’s t tests were applied in (b) and (d). Two-way ANOVA was used in (e) and (f).

Supplementary Figure 5.

**
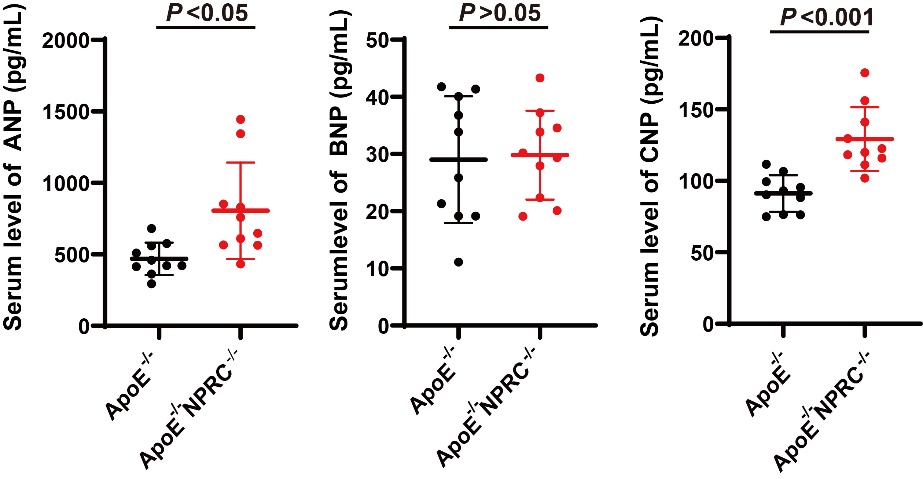
**

**Supplementary Figure 5. Comparison of ANP, BNP and CNP levels between ApoE^-/-^ and ApoE^-/-^NPRC^-/-^ mice.** Quantification of serum levels of ANP, BNP and CNP in ApoE^-/-^ and ApoE^-/-^NPRC^-/-^ mice (n=10 per group). Normal distributions were tested by Shapiro-Wilk method. Unpaired two-tailed Student’s t tests were applied.

Supplementary Figure 6.

**
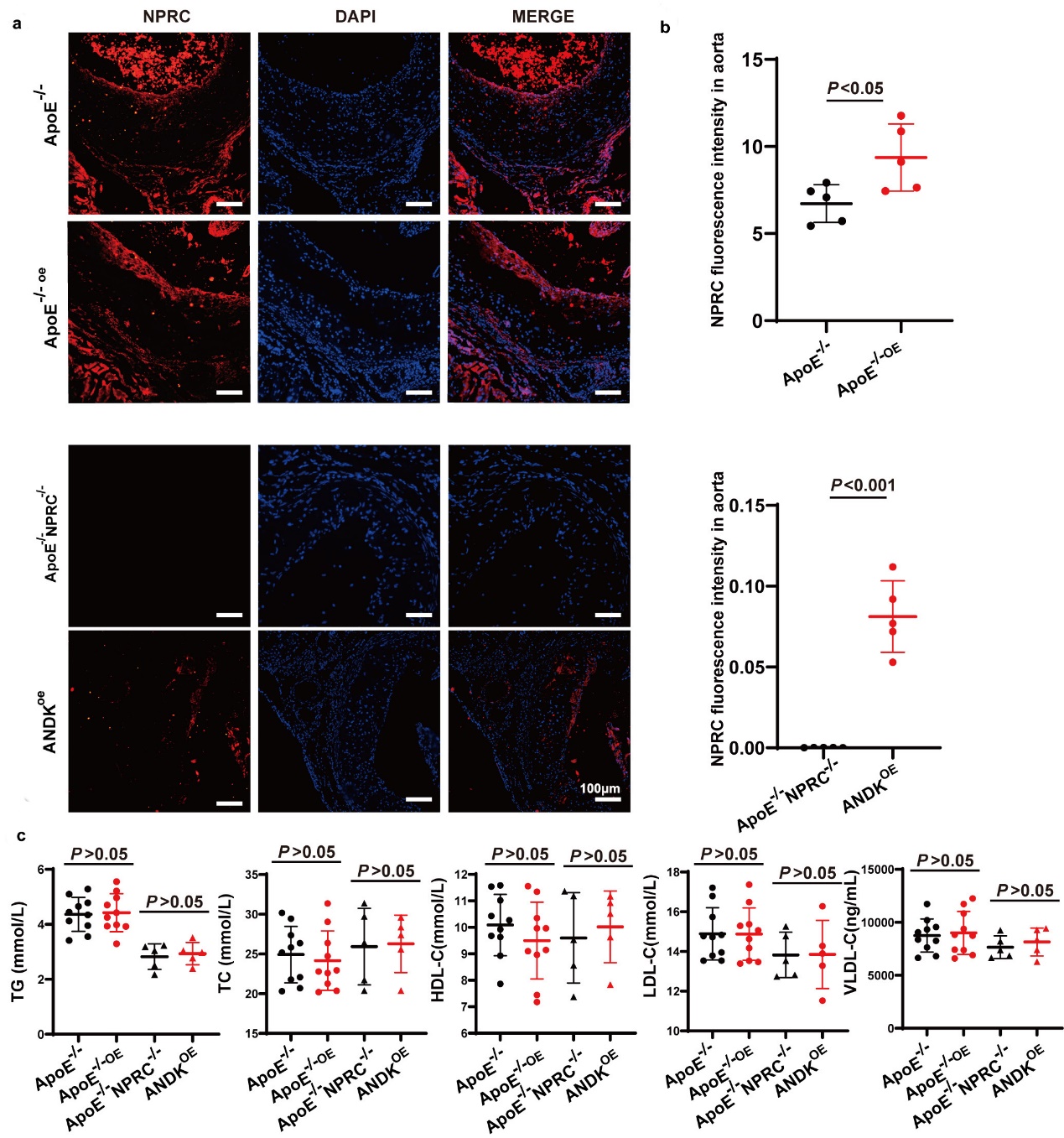
**

**Supplementary Figure 6. NPRC expression and serum lipid and ROS levels in NPRC overexpressed and control mice. (a)** Representative immunofluorescence images of NPRC in aortic roots from ApoE^-/-^, ApoE^-/-OE^, ApoE^-/-^NPRC^-/-^ and ANDK^OE^ mice (scale bar=100μm). **(b)** Quantification of NPRC immunofluorescence intensity in the aortic root from ApoE^-/-^, ApoE^-/-OE^, ApoE^-/-^NPRC^-/-^ and ANDK^OE^ mice (n=5 per group). **(c)** Quantification of serum levels of TG, TC, HDL-C, LDL-C and VLDL-C in ApoE^-/-^, ApoE^-/-OE^, ApoE^-/-^NPRC^-/-^ and ANDK^OE^ mice (n=10 per group). Normal distributions were tested by Shapiro-Wilk method. Unpaired two-tailed Student’s t tests were applied in (b). One-way ANOVA was applied in (c).

Supplementary Figure 7.

**
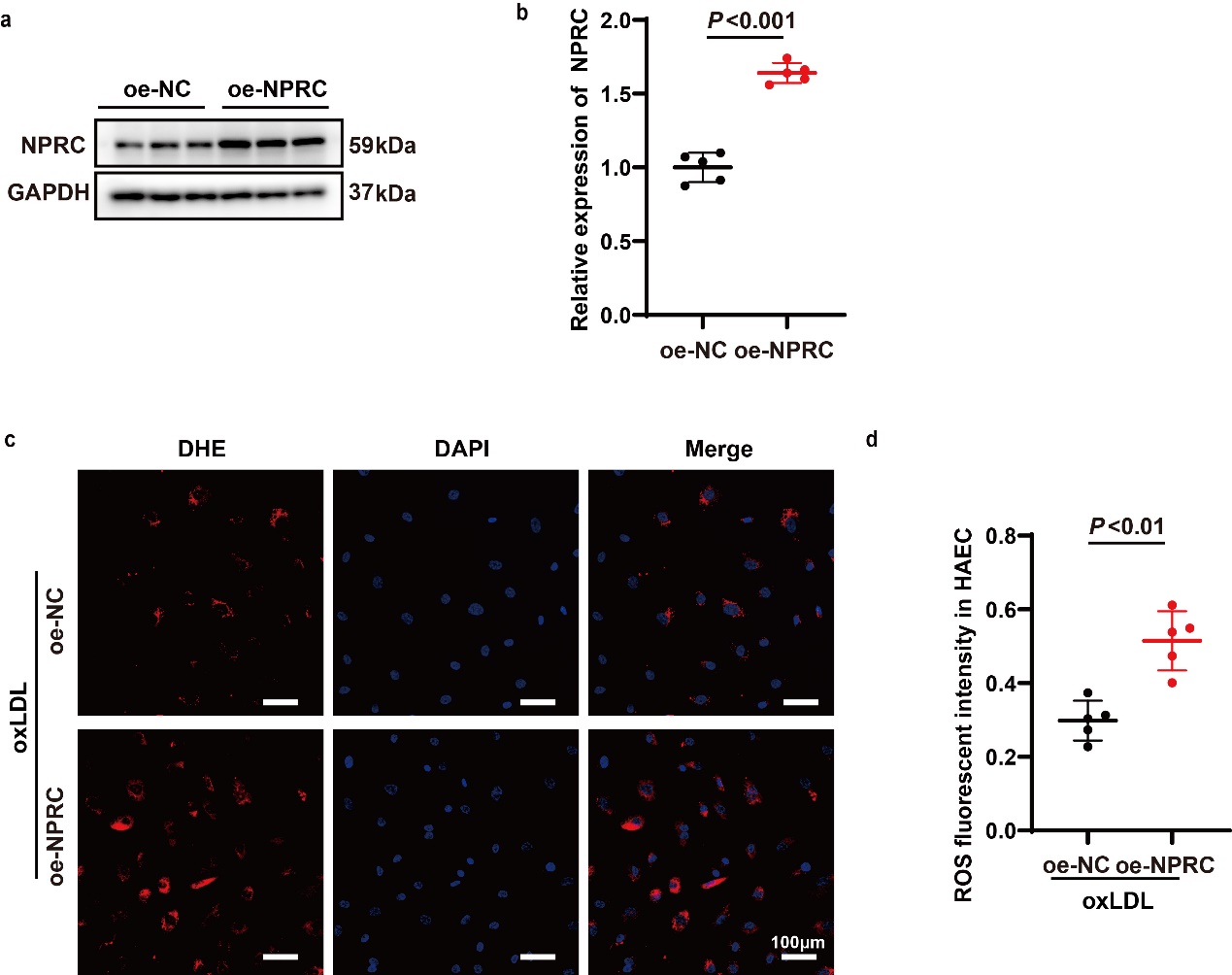
**

**Supplementary Figure 7. NPRC expression and ROS levels in NPRC overexpressed cells.**

**(a)** Representative Western blot images of NPRC expression in oe-NC and oe-NPRC HAECs. **(b)** Quantification of NPRC expression in oe-NC and oe-NPRC HAECs (n=5 per group). **(c)** Representative images of DHE staining of ROS levels in oxLDL- stimulated oe-NC and oe-NPRC HAECs (scale=100μm). **(d)** Quantification of mean fluorescence intensity of ROS in oxLDL-stimulated oe-NC and oe-NPRC HAECs (n=5 per group). Normal distributions were tested by Shapiro-Wilk method. Unpaired two-tailed Student’s t tests were applied in (b) and (d).

**Supplementary Table 1. Reagents and antibodies**

|  | Company | Catalog No. | Concentrations |
| --- | --- | --- | --- |
| NPRC | Sigma-Aldrich | SAB2501867 | IHC/IF: 1:100, western blot: 1:1000 |
| NPRC | Origene | TA501021 | western blot: 1:1000 |
| GAPDH | Cell Signaling Technology | #5174 | western blot: 1:1000 |
| VCAM1 | Abcam | Ab134047 | IHC: 1:100, western blot: 1:1000 |
| ICAM1 | Proteintech | #10831-1  #10020-1 | IHC: 1:600, western blot: 1:1000 |
| TNFα | Cell Signaling Technology | #11948 | IHC: 1:200, western blot: 1:1000 |
| TNFα | Abcam | Ab183218 | western blot:1:1000 |
| IL-6 | Abcam | Ab233706 | western blot: 1:1000 |
| Mcp1 | Abcam | Ab8101 | IHC: 1:200, western blot: 1:1000 |
| IL-6 | Cell Signaling Technology | #12912 | IHC: 1:100, western blot: 1:1000 |
| Moma2 | Abcam | Ab183549 | IHC: 1:200 |
| α-SMA | Cell Signaling Technology | #19245 | IHC: 1:200 |
| P65 | Cell Signaling Technology | #9936 | western blot: 1:1000 |
| pP65 | Cell Signaling Technology | #9936 | western blot: 1:1000 |
| Iκκα | Cell Signaling Technology | #9936 | western blot: 1:1000 |
| pIκκα/β | Cell Signaling Technology | #9936 | western blot: 1:1000 |
| Iκκβ | Cell Signaling Technology | #9936 | western blot: 1:1000 |
| pAkt1 | Cell Signaling Technology | #4060 | western blot: 1:1000 |
| Akt1 | Cell Signaling Technology | #4685 | western blot: 1:1000 |
| Cleaved-caspase3 | Cell Signaling Technology | #9915 | western blot: 1:1000 |
| Caspase3 | Cell Signaling Technology | #9915 | western blot: 1:1000 |
| Cleaved-caspase7 | Cell Signaling Technology | #9915 | western blot: 1:1000 |
| Caspase7 | Cell Signaling Technology | #9915 | western blot: 1:1000 |
| eNOS | Cell Signaling Technology | #32027 | western blot: 1:1000 |
| CREB | Cell Signaling Technology | #9197 | western blot: 1:1000 |
| pCREB | Cell Signaling Technology | #9198 | IHC: 1:200, western blot: 1:1000 |
| VASP | Cell Signaling Technology | #3112 | western blot: 1:1000 |
| pVASP | Cell Signaling Technology | #3111 | western blot: 1:1000 |
| PPARγ | Cell Signaling Technology | #2435 | IHC: 1:200,western blot: 1:1000 |
| PGC1α | Cell Signaling Technology | #2178 | IHC: 1:200, western blot: 1:1000 |
| p-PKA-substrates | Cell Signaling Technology | #9624 | western blot: 1:1000 |
| oxLDL | Xiesheng | #12002 | 100mg/mL |
| H89 | MCE | HY-15979 | 10nM |
| foskolin | MCE | HY-15371 | 20nM |

**Supplementary Table 2. Primer sequences of genes**

| **Gene name** | **Sequence 5’-3’** |
| --- | --- |
| mouse *β-actin* forward | CCACACCCGCCACCAGTTCG |
| mouse *β-actin* reverse | TACAGCCCGGGGAGCATCGT |
| human *β-actin* forward | GGAAATCGTGCGTGACATTAA |
| human *β-actin* reverse | AGGAAGGAAGGCTGGAAGAG |
| mouse *tnf-α* forward | CCCTCACACTCAGATCATCTTCT |
| mouse *tnf-α r*everse | GCTACGACGTGGGCTACAG |
| human *tnf-α* forward | AGAGAAGCAACTACAGACC |
| human *tnf-α* reverse | AGTATGTGAGAGGAAGAGAAC |
| mouse *il-6* forward | AGTTGCCTTCTTGGGACTGA |
| mouse *il-6* reverse | TCCACGATTTCCCAGAGAAC |
| human *il-6* forward | GGCACCTCAGATTGTTGT |
| human *il-6* reverse | TAGTGTCCTAACGCTCATAC |
| mouse *enos* forward | TACTTCCTGGACATCACTTC |
| *mouse enos* reverse | TGTTCGCTGGACTCTTCT |
| mouse *vcam1* forward | GTGGCGGTTATGACTTCA |
| mouse vcam*1* reverse | CTGCGGATGTTCTCAATCT |
| mouse i*cam1* forward | AACGCTGACTTCATTCTCTA |
| mouse *icam1* reverse | CTTCTTGCTTGTGTCTACTG |
| human *nprc* forward | CTACGCCTTCTTCAACATTG |
| human *nprc* reverse | GGACGAGTATGCTTGCTTA |
| mouse PPARγ forward | TGGCGTGTCTTCATAACTC |
| mouse PPARγ reverse | CTGGTATCGGCTCAATAATTC |
| mouse PGC1α forward | TCATCACCTACCGTTACAC |
| mouse PGC1α reverse | AATTGCTTCCGTCCACAA |
| mouse MCP1 forward | TAAAAACCTGGATCGGAACCAAA |
| mouse MCP1 reverse | GCATTAGCTTCAGATTTACGGGT |
| human MCP1 forward | TAGCAGCCACCTTCATTC |
| human MCP1 reverse | TGTTCAAGTCTTCGGAGTT |
